# Strategies for carbon reduction and advertising investments in partially centralized supply chains

**DOI:** 10.1371/journal.pone.0351412

**Published:** 2026-06-16

**Authors:** Weisi Zhang, Jiahao Kong, Wei Zhao, Juanjuan Liu

**Affiliations:** 1 Institute of Logistics Science and Engineering, Shanghai Maritime University, Shanghai, China; 2 School of Economics & Management, Shanghai Maritime University, Shanghai, China; NOVA School of Science and Technology: Universidade Nova de Lisboa Faculdade de Ciencias e Tecnologia, PORTUGAL

## Abstract

Rising concerns about climate change and growing consumer awareness of environmental sustainability have accelerated the adoption of cap-and-trade policies worldwide. This study investigates how different supply chain operation models influence manufacturers’ carbon reduction decisions and retailers’ green advertising strategies. Our analysis reveals that higher carbon trading prices generally stimulate greater emission reduction efforts. However, when both the carbon price and the cost of emission reduction are sufficiently high, further increases in carbon prices may instead weaken firms’ incentives to reduce emissions. We further find that increasing cost-sharing ratios alone does not necessarily improve coordination outcomes. Instead, the effectiveness of coordination depends critically on the structure of cost-sharing. The effectiveness of supply chain coordination hinges on strategic allocation of cost-sharing ratios, specifically, the *RC* model performs better when carbon reduction cost-sharing is low and advertising cost-sharing is high. When carbon reduction cost-sharing is high, the *MC* model is preferred under low advertising cost-sharing, whereas the *DC* model becomes more effective when advertising cost-sharing is high.

## 1. Introduction

### 1.1. Background and motivation

As low-carbon and green development initiatives continue to gain global attention, sustainable supply chain management has become increasingly critical. This requires manufacturers to adopt eco-friendly production and retailers to follow sustainable practices [1,2]. As a result, green supply chain management, which includes carbon reduction, green advertising, and cost-sharing contracts [3], has drawn significant attention from governments, consumers, and competitors.

To mitigate carbon emissions and address climate change, many countries have implemented market-based regulatory instruments. Among them, the cap-and-trade mechanism has become one of the most widely adopted policy tools [4,5]. Under this system, firms are allocated emission quotas and are allowed to trade carbon allowances in a regulated market, thereby internalizing environmental externalities and incentivizing emission reduction behaviors [6].

In parallel, green advertising has emerged as an effective strategy for retailers to influence consumer preferences and stimulate demand for low-carbon products. By enhancing consumer awareness of environmental attributes, retailers can translate sustainability concerns into purchasing behavior. For example, firms such as ERKE have adopted eco-friendly materials in product design, reflecting the growing integration of sustainability into marketing strategies.

In green supply chains, coordination between manufacturers and retailers plays a crucial role in improving both environmental and economic performance [7]. Empirical studies have shown that cooperation mechanisms can enhance supply chain efficiency and sustainability outcomes [8]. Among these mechanisms, cost-sharing contracts are widely used to align incentives and improve joint decision-making between supply chain members [9].

However, firms face a fundamental trade-off between environmental investment and economic cost. Increasing carbon reduction efforts or expanding green advertising both require additional expenditures, while their effectiveness depends on market conditions such as consumer environmental awareness and regulatory intensity. This makes it necessary to investigate how different coordination structures influence optimal carbon reduction and advertising decisions under varying cost-sharing arrangements.

### 1.2. Research gap

Existing research on supply chain emission reduction under cost-sharing contracts has provided valuable insights into coordination mechanisms and firms’ environmental strategies. Prior studies have examined how different contract forms, such as revenue-sharing, wholesale pricing, and cost-sharing arrangements, influence emission reduction decisions under various market settings [10,11]. However, these studies typically focus on isolated decision dimensions or specific coordination structures.

Carbon trading price fluctuations, government policy changes, and shifting consumer preferences significantly influence supply chain emission reduction decisions and cost-sharing approaches. Developing adaptive emission reduction models and evaluating their practical effectiveness represent urgent research priorities. In particular, how different operational structures (centralized, decentralized, and partially centralized systems) affect the strategic interaction between carbon reduction and green advertising remains underexplored [12,13].

Under the cap-and-trade policy, governments allocate initial carbon quotas to manufacturers through grandfathering provisions. Manufacturers conduct emission reduction activities and participate in carbon trading while retailers invest in green advertising. This research compares fully centralized and decentralized supply chain models while examining cost-sharing contracts for coordination. Understanding optimal decisions across different models and their influencing factors remains essential.

Therefore, this study aims to fill this gap by developing a unified analytical framework that compares centralized, decentralized, and partially centralized supply chain structures, and systematically examines how different cost-sharing mechanisms shape manufacturers’ carbon reduction decisions and retailers’ green advertising strategies under cap-and-trade regulation.

### 1.3. Major findings

Based on the discussions above, this study addresses three key research questions: (1) How do carbon trading prices affect carbon reduction rates? (2) How do consumer preferences for low-carbon products and advertising effectiveness influence carbon reduction and advertising strategies under different cost-sharing ratios? (3) Which supply chain coordination models prove most effective under specific ratios of carbon reduction and green advertising cost-sharing?

The major findings of this study reveal several important insights. Generally, higher carbon trading prices are associated with greater carbon reduction efforts by manufacturers. However, when both carbon trading prices and carbon reduction cost coefficients reach high levels, additional increases in carbon prices may paradoxically lead to reduced emission reduction efforts. The analysis further shows that as consumer preferences shift toward low-carbon products and advertising effectiveness improves, both the optimal carbon reduction rates and advertising intensity tend to increase. It is noteworthy that simply raising cost-sharing ratios does not automatically guarantee stronger carbon reduction or advertising outcomes. Regarding supply chain coordination strategies, the distribution of cost-sharing ratios plays a decisive role. The *RC* model emerges as the preferred choice when the cost-sharing ratio for carbon reduction is relatively low while the ratio for green advertising is high. In cases where the carbon reduction cost-sharing ratio is high, the selection depends on the green advertising ratio: the *MC* model is optimal when advertising cost-sharing is low, whereas the *DC* model proves more effective when advertising cost-sharing is high.

This paper is organized as follows: Section 2 reviews relevant literature and outlines our contributions to the field. Section 3 presents a mathematical formulation and analysis of centralized, decentralized, and cost-sharing contract issues. Section 4 derives optimal solutions based on a Hessian matrix analysis, discussing how different parameters affect the strategic choices of participants. Section 5 uses numerical simulations to validate the study’s findings and discusses the managerial implications for supply chain managers. The study concludes in Section 6, with all proofs included in the S1 Appendix.

## 2. Literature review

To effectively tackle the challenges posed by carbon emissions in supply chains, enterprises are adopting innovative methods to enhance the environmental sustainability of their operations. Accordingly, this paper reviews literature related to consumers’ green preferences, green supply chain decisions, and cost-sharing contracts.

### 2.1. Consumer green preferences

The impact of consumer green preferences on green supply chains is a critical topic, especially in a global context increasingly focused on sustainable development and environmental protection. The environmental attributes of products and the quality of advertising have become key factors influencing consumer purchasing decisions [14,15]. Consumers tend to favor environmentally friendly products, prompting enterprises to source sustainable materials and employ eco-friendly production processes, thereby driving the green transformation of the entire supply chain [16].

Other researchers are more interested in the impact of consumer green preferences on green supply chain decisions. Findings suggest that emission reduction management within supply chains can align well with consumer preferences, enhancing business operational benefits. The lag in emission reduction technology and consumers’ preference for low-carbon options positively influence manufacturers’ level of carbon emission transfers [17]. Research into the optimal channels of green supply chains reveals that, regardless of the type of channel structure involved, consumers’ awareness of environmental issues encourages manufacturers to enhance the green credentials of their products. However, in cases where retailers operate offline and manufacturers online, the proportion of online consumers positively influences the greening of products. Conversely, in scenarios where retailers operate online and manufacturers offline, this influence is negative [18].

### 2.2. Green supply chain decisions

Green supply chain operational decisions have recently attracted widespread attention from both academic researchers and practitioners. Decision-making within enterprises in a low-carbon environment inevitably impacts the sustainability of supply chains. An increasing body of research is focusing on operational decisions in green supply chains, including pricing, production, carbon reduction, and green advertising [19]. From the perspectives of production and pricing, the use of the Stackelberg game model has yielded optimal production and pricing strategies for members of the chain in both centralized and decentralized cases [20]. Liu et al. [5] explored the joint production and pricing problems of manufacturers under cap-and-trade and carbon tax policies. Their findings indicate that emission caps and emission pricing significantly affect optimal production decisions and profitability. Environmental departments can regulate environmental policies and impose emission caps.

In the domains of emission reduction and green advertising, numerous studies have shown that engaging in carbon reduction and green advertising can bring profits or policy subsidies to members of the supply chain [21,22]. Tao et al. [18] constructed a game model involving manufacturers and retailers, using a low-carbon supply chain channel as an example, to demonstrate how contract design can optimize low-carbon supply chain management decisions and enhance supply chain performance. Hsieh et al. [23] investigate the interactive dynamics of a green supply chain under asymmetric platform competition, where the incumbent platform offers blockchain services while the new platform does not. Considering targeted advertising and consumer performance information in a new context, the role of targeted advertising in enhancing retailers’ green advertising efforts was explored.

### 2.3. Cost-sharing contracts

Research in the area of cost-sharing contracts has been extensive, focusing on diverse contractual scenarios within supply chains. A significant aspect of this research involves the construction of collaborative models that include a retailer and two manufacturers, exploring two distinct contractual scenarios: both manufacturers operating under cost-sharing agreements, and one manufacturer under a cost-sharing contract while the other is under a wholesale pricing contract [24–26]. The findings highlight that cost-sharing is effectively utilized not only for sharing costs between manufacturers and retailers but also for enhancing supply chain cooperation by involving retailers in sharing manufacturers’ costs. Utilizing differential game theory, the research has evaluated various cost-sharing strategies, such as scenarios where manufacturers do not share any costs, those where they share costs related to emission reductions, and others where retailers share service-related costs. The results from these studies indicate that reciprocal cost-sharing contracts can be advantageous for the entire supply chain and its constituents [3,27,28].

Additionally, recent studies have introduced innovative bargaining mechanisms within cost-sharing contracts to examine their effects on green supply chains [27], comparing outcomes with cartelization, standard cost-sharing contracts, and non-cooperative scenarios [8]. This line of research also includes analysis of decentralized versus centralized cost-sharing mechanisms, clearly identifying the conditions necessary to achieve mutually beneficial outcomes [18]. Cao et al. [29] expand the application of blockchain technology, game models that treat the usage level of blockchain technology as an exogenous variable, and those that only address the design of single-type cost-sharing contracts. This enhances the understanding of the strategic applications of cost-sharing strategies and their significant impact on fostering supply chain cooperation.

### 2.4. Summary

Supply chain emission reduction remains a central topic in the literature, with extensive research examining the role of regulatory instruments such as carbon taxes and emission quotas in shaping firms’ environmental decisions. Existing studies have provided important insights into manufacturers’ emission reduction efforts and the role of coordination mechanisms, particularly cost-sharing contracts, in improving supply chain performance.

At the same time, prior research has begun to explore demand-side factors, such as consumer environmental preferences, although relatively limited attention has been paid to the role of retailers’ green advertising in influencing market demand and interacting with emission reduction decisions. In addition, while cost-sharing contracts have been widely studied, most works consider either emission reduction or marketing decisions in isolation, rather than examining their joint effects within a unified framework.

Building on this stream of literature, this paper makes several contributions. First, rather than focusing on government subsidies, we analyze manufacturers’ decisions under cap-and-trade regulation, which better reflects real-world carbon market mechanisms. Second, we incorporate retailers’ green advertising into the analysis and examine its interaction with carbon reduction decisions, thereby extending existing studies on demand-side sustainability drivers. Third, we investigate supply chain coordination under partial centralization, providing a more flexible structure beyond the traditional fully centralized and decentralized models. By integrating these elements, this paper offers a more comprehensive framework for analyzing the interplay between carbon reduction, advertising, and coordination strategies.

## 3. Problem formulation

### 3.1. Model description

This study primarily analyzes the challenges of emission reduction by manufacturer and green advertising by retailer under cap-and-trade policy. The supply chain consists of one manufacturer, one retailer, and the government. According to grandfathering provisions [26], the government allocates initial carbon quotas to manufacturer. If the initial carbon quota is less than (or greater than) the actual carbon emissions, the deficit (or surplus) can be bought (or sold) in the carbon trading market. Building on the models of complete centralization (*SC*) and decentralization (*DC*), this study also considers scenarios of commonly adopted partial centralization. The focus is on cost-sharing ratios, specifically how the retailer can share in manufacturer’s carbon reduction costs (*RC*) and the manufacturer in retailer’s green advertising costs (*MC*) through cost-sharing contract mechanisms. Considering consumer preferences for low-carbon products, the study investigates the impact of green advertising on demand stimulation and develops combined strategies for manufacturer’s carbon reduction and retailer’s green advertising.

### 3.2. Assumption

Based on the model description, to align the model with general real-world scenarios while enabling rational analysis and effective conclusions, the following assumptions are made:

(1) The demand for the product is primarily influenced by the product’s price, consumer preferences for the product’s environmental attributes, and the retailer’s green advertising efforts. Consumer preferences are mainly reflected in their sensitivity to the product and the retailer’s green advertising efforts. The market demand function for the product can thus be expressed as D=a−p+te+zγ [27]. Here, a represents the initial market demand for the product, p denotes the retail price, e is the manufacturer’s carbon reduction rate, and γ represents the retailer’s green advertising efforts, with t and z being the demand sensitivity coefficients for the product’s carbon reduction rate and green advertising efforts, respectively [30,31]. The use of a linear demand function is consistent with standard practice in the supply chain and game theory literature. It captures the negative relationship between price and market demand while ensuring analytical tractability, allowing a clear examination of strategic interactions between the manufacturer and the retailer.
(2) Considering cap-and-trade policy, the government allocates a carbon emission allowance to the manufacturer, denoted as $E_{g}$ [32,33], and $P_{c}$ represents the price of carbon on the carbon trading market [34]. The trading of carbon quotas takes place in a standardized market where emissions allowances serve as the commodity, established through a cap system. The manufacturer can sell its surplus emission rights or purchase others’ emission rights in the carbon trading market to meet its own emission requirements. The specific formula is $P_{c}((\text{1}-e)D-E_{g})$, where the enterprise controls its own carbon emissions through its reduction efforts. Here, (1−e)D represents the total carbon emissions; if $(\text{1}-e)D-E_{g}$ is negative, it indicates that the enterprise has a surplus of carbon emissions, which can be sold [35].
(3) To protect the environment and enhance the green credentials of their products, manufacturers will increase their carbon reduction rates during production, and retailers will similarly enhance their advertising efforts during the selling process. Assume the cost of carbon reduction for the manufacturer is $\frac{ke^{\text{2}}}{\text{2}}$ [27,28,36], where k is the coefficient representing the cost of carbon reduction for the manufacturer. The cost of green advertising for the retailer is $\frac{v\gamma^{\text{2}}}{\text{2}}$ [32,37], where v represents the coefficient for the retailer’s green advertising effort. The quadratic cost functions for emission reduction and advertising efforts are adopted to represent increasing marginal costs, a widely accepted assumption in related studies. This formulation reflects the rising difficulty and cost of additional investments, guarantees the concavity of profit functions, and aligns with realistic production and marketing conditions in green supply chains. The notations and parameters used throughout this paper are summarized in Table 1.

**Table 1:** Notations.

Parameter	Definition description
a	Potential market demand
t	Consumer low-carbon preference
z	Advertising effectiveness
k	Cost coefficient for emission reduction efforts
v	Cost coefficient for advertising efforts
θ	Sharing ratio of carbon reduction costs (0<θ<1)
δ	Sharing ratio of green advertising costs (0<δ<1)
$P_{c}$	Unit carbon trading price
$E_{g}$	Initial carbon emission quota
Decision variables	
$w^{i}$	Wholesale price (*i* = *SC*, *DC*, *RC*, *MC*)
$p^{i}$	Retail price (*i* = *SC*, *DC*, *RC*, *MC*)
$e^{i}$	Carbon reduction rate (*i* = *SC*, *DC*, *RC*, *MC*)
$\gamma^{i}$	Advertising effort (*i* = *SC*, *DC*, *RC*, *MC*)
Profit	
$\pi_{s}$	Profit of the supply chain under complete centralization
$\overset{i}{\underset{m}{\pi }}$	Manufacturer profit (*i* = *DC*, *RC*, *MC*)
$\overset{i}{\underset{r}{\pi }}$	Retailer profit (*i* = *DC*, *RC*, *MC*)
Threshold parameter	
$\theta^{h}$	Hessian matrix constraints
$\overset{i}{\underset{g}{\theta }},\;\overset{i}{\underset{g}{\delta }}$	Profit comparison thresholds across different modes (i=m, r and g=1, 2, 3)

### 3.3. Sequence of decisions

**Fig 1:**
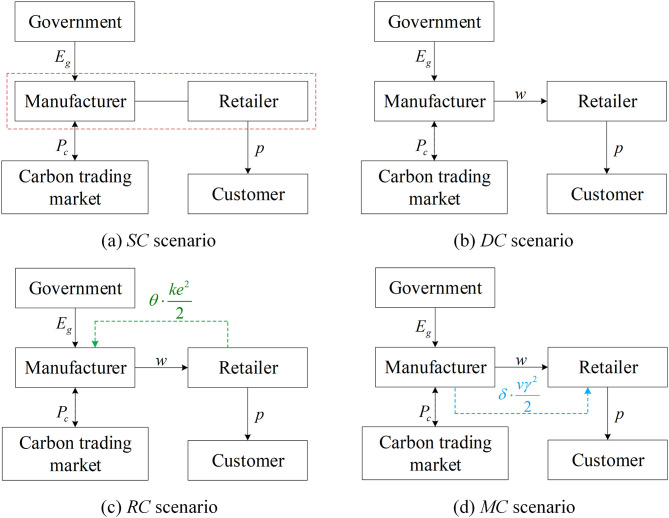
Model structures under different scenarios.

Specific models for different scenarios are depicted in Fig 1.

**Fig 2:**
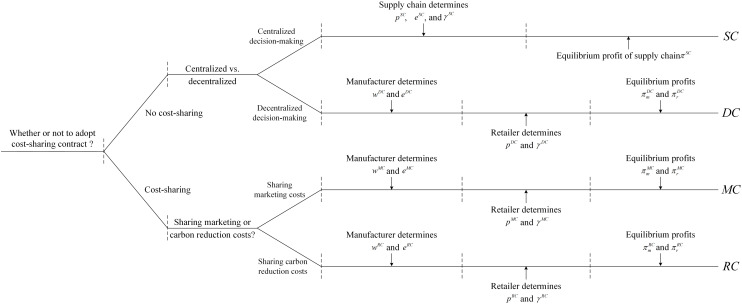
Sequence of events.

In green sustainable supply chains, the typical structure involves manufacturers as Stackelberg leaders and retailers as followers. The process unfolds through three main stages, as shown in Fig 2.

*Stage one*: Supply chain participants consider whether to introduce cost-sharing contract to facilitate partially centralized supply chain structure.

*Stage two*: If no cost-sharing contract is introduced, the supply chain must choose between complete centralization or complete decentralization. Conversely, with cost-sharing contract, considerations include whether retailers share the costs of manufacturers’ carbon reduction or manufacturers share the costs of retailers’ green advertising.

*Stage three*: Operational decisions are made as follows: Firstly, manufacturers set the unit wholesale price and carbon reduction rate during production (if opting for *DC*, *RC*, *MC*), or the supply chain entity decides on the product’s unit price, carbon reduction rate, and green advertising effort level (if opting for *SC*). Secondly, retailers determine the retail price and green advertising effort level (if opting for *DC*, *RC*, *MC*). Finally, each channel decides on the order quantity, with all enterprises obtaining corresponding profits.

## 4. Equilibrium analysis

### 4.1. Complete centralization (SC)

In centralized decision-making scenarios, manufacturer and retailer collaborate through vertical integration to jointly pursue the maximization of the total the supply chain profit. The supply chain determines the optimal retail price, carbon emission reduction rate, and low-carbon marketing effort level based on the objective of maximizing profits.


$$\begin{array}{c} \pi_{s}(p,e,\gamma )=pD-P_{c}((\text{1}-e)D-E_{g})-\frac{ke^{\text{2}}}{\text{2}}-\frac{v\gamma^{\text{2}}}{\text{2}} \end{array}$$ (1)

The total profit of the supply chain comprises four parts: the first part is the revenue from product sales, the second part is the cost associated with carbon trading, and the third and fourth parts are the costs of carbon reduction and low-carbon marketing efforts, respectively. The equilibrium outcomes under centralized decision-making are presented in Lemma 1.

**Lemma 1.** *Under the scenario SC, the equilibrium retail price, carbon reduction rate, advertising effort, and the supply chain profit are denoted as* $p^{SC}=\frac{P_{c}v(t+P_{c})(a+t)-P_{c}z^{\text{2}}k-kv(a+P_{c})}{kz^{\text{2}}+v(t+P_{c}{)}^{\text{2}}-\text{2}kv}$*,* $e^{SC}=\frac{v(P_{c}-a)(t+P_{c})}{kz^{\text{2}}+v(t+P_{c}{)}^{\text{2}}-\text{2}kv}$*,* $\gamma^{SC}=\frac{kz(P_{c}-a)}{kz^{\text{2}}+v(t+P_{c}{)}^{\text{2}}-\text{2}kv}$*, and* $\pi_{s}(p,e,\gamma )=\frac{\text{2}P_{c}E_{g}kz^{\text{2}}+\text{2}P_{c}E_{g}v(t+P_{c}{)}^{\text{2}}-\text{4}P_{c}E_{g}kv-kv(a-P_{c}{)}^{\text{2}}}{\text{2}kz^{\text{2}}+\text{2}v(t+P_{c}{)}^{\text{2}}-\text{4}kv}$*, respectively*.

### 4.2. Complete decentralization (DC)

In decentralized decision-making within the supply chain, members make decisions to maximize their own interests. Based on the Stackelberg game model, where the manufacturer acts as the leader and the retailer as the follower, the specific game process is as follows: the manufacturer first sets the unit wholesale price and the carbon reduction rate during production. Subsequently, the retailer determines the retail price and the level of green advertising efforts. Using a backward induction method, the optimal solutions for the decision variables are obtained under this game scenario. The profit functions for the manufacturer and retailer under decentralized decision-making are as follows:


$$\begin{array}{c} \overset{DC}{\underset{m}{\pi }}(w,e)=wD-\frac{ke^{\text{2}}}{\text{2}}-P_{c}\;((\text{1}-e)D-E_{g})\;\; \end{array}$$ (2)

$$\begin{array}{c} \overset{DC}{\underset{r}{\pi }}(p,\gamma )=(p-w)D-\frac{v\gamma^{\text{2}}}{\text{2}} \end{array}$$ (3)

According to the first-order conditions, the optimal decisions in the supply chain under decentralized decision-making scenarios can be represented as Lemma 2.

**Lemma 2.** *Under the scenario DC, the optimal wholesale price and carbon reduction rate for the manufacturer, the optimal retail price and green advertising effort level for the retailer, and the optimal profits for each party in the supply chain are respectively:* $w^{DC}=\frac{k(P_{c}+a)(\text{2}v-z^{\text{2}})-P_{c}v(P_{c}+t)(t+a)}{\text{2}k(\text{2}v-z^{\text{2}})-v(t+P_{c}{)}^{\text{2}}}$*,* $e^{DC}=\frac{v(a-P_{c})(t+P_{c})}{\text{2}k(\text{2}v-z^{\text{2}})-v(t+P_{c}{)}^{\text{2}}}$*,* $p^{DC}=\frac{k(P_{c}+a)(v-z^{\text{2}})-P_{c}v(a+t)(P_{c}+t)+\text{2}kva}{\text{2}k(\text{2}v-z^{\text{2}})-v(t+P_{c}{)}^{\text{2}}}$*,* $\gamma^{DC}=\frac{kz(a-P_{c})}{\text{2}k(\text{2}v-z^{\text{2}})-v(t+P_{c}{)}^{\text{2}}}$*,* $\overset{DC}{\underset{m}{\pi }}(w,e)=\frac{kv(a-P_{c}{)}^{\text{2}}+\text{4}P_{c}E_{g}k(\text{2}v-z^{\text{2}})-\text{2}P_{c}E_{g}v(t+P_{c}{)}^{\text{2}}}{\text{4}k(\text{2}v-z^{\text{2}})-\text{2}v(t+P_{c}{)}^{\text{2}}}$*,* $\overset{DC}{\underset{r}{\pi }}(p,\gamma )=\frac{k^{\text{2}}v(a-P_{c}{)}^{\text{2}}(\text{2}v-z^{\text{2}})}{\text{2}{\left(\text{2}k(\text{2}v-z^{\text{2}})-v(t+P_{c}{)}^{\text{2}}\right)}^{\text{2}}}$.

Building on Lemma 2, further analysis was conducted on the impact of the unit carbon price and initial carbon quotas on carbon reduction, as well as the influence of consumer preferences on supply chain decisions. Corollaries 1 present the conclusions.

**Corollary 1.** *Under the scenario DC, the impact of consumer preferences on supply chain decisions is characterized as follows*:

(*i*) $\frac{\partial e^{DC}}{\partial t}>\text{0}$*,* $\frac{\partial \overset{DC}{\underset{m}{\pi }}}{\partial t}>\text{0}$*.* (*ii*) $\frac{\partial \gamma^{DC}}{\partial z}>\text{0}$*,* $\frac{\partial \overset{DC}{\underset{r}{\pi }}}{\partial z}>\text{0}$*.*

According to Corollary 1, in decentralized scenarios, increased consumer sensitivity to product carbon reduction and green advertising efforts enhances both carbon reduction rates and intensity of green advertising efforts.

Corollary 1 indicates that as consumer sensitivity to carbon reduction increases, supply chain enterprises can achieve higher profits by aligning with this preference. Therefore, supply chain enterprises and governments should intensify low-carbon and environmental protection campaigns to enhance consumer preferences for low-carbon products, which will aid in building environmentally friendly society and promote economic development.

### 4.3. Partial centralization

#### 4.3.1. Retailer sharing carbon reduction costs (RC).

In the scenario where retailer shares carbon emission reduction costs with manufacturer, retailer facilitates increased carbon emission reduction rates in products through cost-sharing. This approach enhances consumer preference for products sold and boosts their competitiveness in the market. For example, Walmart initiated “Project Gigaton” to motivate manufacturer to increase investments in producing low-carbon products, aiming to assist upstream enterprises in achieving carbon reduction target of one million tons by 2030. Under this partial centralization, retailer’s share of carbon reduction cost is denoted by θ (0<θ<1), while manufacturer bears remaining (1−θ) proportion of carbon reduction costs [3]. In this scenario where retailer shares manufacturer’s reduction costs, profit function for both manufacturer and retailer are as follows:


$$\begin{array}{c} \overset{RC}{\underset{m}{\pi }}(w,e)=wD-(\text{1}-\theta )\frac{ke^{\text{2}}}{\text{2}}-P_{c}((\text{1}-e)D-E_{g}) \end{array}$$ (4)

$$\begin{array}{c} \overset{RC}{\underset{r}{\pi }}(p,\gamma )=(p-w)D-\frac{v\gamma^{\text{2}}}{\text{2}}-\theta \frac{ke^{\text{2}}}{\text{2}} \end{array}$$ (5)

According to the first-order conditions, the optimal decisions in model *RC* decision-making scenario for the supply chain can be expressed as Lemma 3.

**Lemma 3.** *Under the scenario RC, the optimal wholesale price and carbon reduction rate for the manufacturer, optimal retail price and green advertising effort level for the retailer, and optimal profits for each party in the supply chain are given by* $w^{RC}=\frac{aP_{c}(P_{c}+t)v+P_{c}t(P_{c}+t)v+ak(\text{2}v-z^{\text{2}})(-\text{1}+\theta )+kP_{c}(\text{2}v-z^{\text{2}})(-\text{1}+\theta )}{{\left(P_{c}+t\right)}^{\text{2}}v+\text{2}k(\text{2}v-z^{\text{2}})(-\text{1}+\theta )}$*,* $e^{RC}=-\frac{(a-P_{c})(P_{c}+t)v}{{\left(P_{c}+t\right)}^{\text{2}}v+\text{2}k(\text{2}v-z^{\text{2}})(-\text{1}+\theta )}$*,* $p^{RC}=\frac{aP_{c}(P_{c}+t)v+P_{c}t(P_{c}+t)v+kP_{c}(v-z^{\text{2}})(-\text{1}+\theta )+ak(\text{3}v-z^{\text{2}})(-\text{1}+\theta )}{{\left(P_{c}+t\right)}^{\text{2}}v+\text{2}k(\text{2}v-z^{\text{2}})(-\text{1}+\theta )}$*,* $\gamma^{RC}=\frac{k(a-P_{c})z(-\text{1}+\theta )}{{\left(P_{c}+t\right)}^{\text{2}}v+\text{2}k(\text{2}v-z^{\text{2}})(-\text{1}+\theta )}$*,* $\overset{RC}{\underset{m}{\pi }}(w,e)=\frac{\begin{array}{c} P_{c}(\text{2}E_{g}(pc+t{)}^{\text{2}}v+kP_{c}v(-\text{1}+\theta )+\\ \text{4}E_{g}k(\text{2}v-z^{\text{2}})(-\text{1}+\theta ))+a^{\text{2}}kv(-\text{1}+\theta )-\text{2}akP_{c}v(-\text{1}+\theta ) \end{array}}{\text{2}{\left(P_{c}+t\right)}^{\text{2}}v+\text{4}k(\text{2}v-z^{\text{2}})(-\text{1}+\theta )}$*, and* $\overset{RC}{\underset{r}{\pi }}(p,\gamma )=\frac{k{\left(a-P_{c}\right)}^{\text{2}}v(k(\text{2}v-z^{\text{2}}){\left(-\text{1}+\theta \right)}^{\text{2}}-{\left(P_{c}+t\right)}^{\text{2}}v\theta )}{\text{2}{\left({\left(P_{c}+t\right)}^{\text{2}}v+\text{2}k(\text{2}v-z^{\text{2}})(-\text{1}+\theta )\right)}^{\text{2}}}$.

Building on Lemma 3, further analysis was conducted to assess the impacts of the unit carbon price and initial carbon quotas on carbon reduction, as well as the influence of consumer preferences on supply chain decisions.

**Corollary 2.** *Under the scenario RC, the impact of consumer preferences on supply chain decisions is as follows*:

(*i*) $\frac{\partial e^{RC}}{\partial t}>\text{0}$; $\frac{\partial \overset{RC}{\underset{m}{\pi }}}{\partial t}>\text{0}$; (*ii*) $\frac{\partial \gamma^{RC}}{\partial z}>\text{0}$; $\frac{\partial \overset{RC}{\underset{r}{\pi }}}{\partial z}>\text{0}$*, when* $\theta \leq \frac{\text{1}}{\text{5}}$ *or when* $k^{RC}>\frac{{\left(P_{c}+t\right)}^{\text{2}}v(\text{5}\theta -\text{1})}{\text{2}\left(\text{2}v-z^{\text{2}}\right)(\text{1}-\theta {)}^{\text{2}}}>\frac{{\left(P_{c}+t\right)}^{\text{2}}v}{\text{2}\left(\text{2}v-z^{\text{2}}\right)(\text{1}-\theta )}$ *and* $\theta >\frac{\text{1}}{\text{3}}$ *or when* $k^{RC}>\frac{{\left(P_{c}+t\right)}^{\text{2}}v}{\text{2}\left(\text{2}v-z^{\text{2}}\right)(\text{1}-\theta )}>\frac{{\left(P_{c}+t\right)}^{\text{2}}v(\text{5}\theta -\text{1})}{\text{2}\left(\text{2}v-z^{\text{2}}\right)(\text{1}-\theta {)}^{\text{2}}}$ *and* $\frac{\text{1}}{\text{5}}<\theta \leq \frac{\text{1}}{\text{3}}$; $\frac{\partial \overset{RC}{\underset{r}{\pi }}}{\partial z}<\text{0}$*, when* $\frac{{\left(P_{c}+t\right)}^{\text{2}}v(\text{5}\theta -\text{1})}{\text{2}\left(\text{2}v-z^{\text{2}}\right)(\text{1}-\theta {)}^{\text{2}}}>k^{RC}>\frac{{\left(P_{c}+t\right)}^{\text{2}}v}{\text{2}\left(\text{2}v-z^{\text{2}}\right)(\text{1}-\theta )}$ *and* $\theta >\frac{\text{1}}{\text{3}}$.

According to Corollary 2, under the scenario where the retailer shares the manufacturer’s emission reduction costs, the manufacturer’s carbon reduction rate, the retailer’s green advertising efforts, and the manufacturer’s profits all increase with the cost-sharing ratio.

Corollary 2 indicates that a higher sharing ratio may impose greater burdens on the retailer, thereby reducing its profits. While sharing the manufacturer’s costs can benefit the manufacturer, the retailer’s profits will only increase with the cost-sharing ratio when the carbon reduction costs are relatively low and the cost-sharing ratio is within a lower range, benefiting both the manufacturer and the retailer.

**Corollary 3.** *Under the scenario RC, the impact of the cost-sharing ratio* θ *on supply chain members’ decisions is as follows*:

$\frac{\partial \overset{RC}{\underset{m}{\pi }}}{\partial \theta }>\text{0}$; $\frac{\partial \overset{RC}{\underset{r}{\pi }}}{\partial \theta }>\text{0}$*, when* $\frac{{\left(P_{c}+t\right)}^{\text{2}}v}{\text{4}\left(\text{2}v-z^{\text{2}}\right)\theta }>k^{RC}>\frac{{\left(P_{c}+t\right)}^{\text{2}}v}{\text{2}\left(\text{2}v-z^{\text{2}}\right)(\text{1}-\theta )}$ *and* $\theta \leq \frac{\text{1}}{\text{3}}$; $\frac{\partial \overset{RC}{\underset{r}{\pi }}}{\partial \theta }<\text{0}$*, when* $k^{RC}>\frac{{\left(P_{c}+t\right)}^{\text{2}}v}{\text{4}\left(\text{2}v-z^{\text{2}}\right)\theta }>\frac{{\left(P_{c}+t\right)}^{\text{2}}v}{\text{2}\left(\text{2}v-z^{\text{2}}\right)(\text{1}-\theta )}$.

According to Corollary 3, when the retailer shares the manufacturer’s costs, the manufacturer’s carbon reduction rate, the retailer’s green advertising efforts, and the manufacturer’s profits all increase with the cost-sharing ratio.

Corollary 3 indicates that while sharing the manufacturer’s costs can benefit the manufacturer, the retailer’s profits will only increase with the cost-sharing ratio when the emission reduction costs are relatively low and the cost-sharing ratio is within a lower range. In such cases, both the manufacturer and the retailer benefit.

#### 4.3.2. Manufacturer sharing advertising costs (MC).

Under the scenario where the manufacturer shares the costs of the retailer’s green advertising, the manufacturer enhances its brand image by sharing costs, ensuring that its low-carbon products are widely recognized through the retailer’s advertising efforts. For instance, Intel once paid over a billion dollars to retailers for product promotion. In the specific model, the manufacturer’s cost-sharing ratio is denoted by δ (0<δ<1), while the retailer bears the remaining (1−δ) proportion of the costs. Based on the Stackelberg game model, with the manufacturer as the leader and the retailer as the follower, the optimal solutions for the decision variables in this game scenario are derived using a backward-solving approach. Under the scenario where the manufacturer shares the costs of the retailer’s green advertising, the profit functions for the manufacturer and the retailer are as follows:


$$\begin{array}{c} \overset{MC}{\underset{m}{\pi }}(w,e)=wD-\frac{ke^{\text{2}}}{\text{2}}-P_{c}((\text{1}-e)D-E_{g})-\delta \frac{v\gamma^{\text{2}}}{\text{2}} \end{array}$$ (6)

$$\begin{array}{c} \overset{MC}{\underset{r}{\pi }}(p,\gamma )=(p-w)D-(\text{1}-\delta )\frac{v\gamma^{\text{2}}}{\text{2}} \end{array}$$ (7)

According to the first-order conditions, the optimal decisions in a decentralized decision-making scenario for the supply chain can be expressed as Lemma 4.

**Lemma 4.** *Under the scenario MC, the optimal wholesale price and product carbon reduction rate for the manufacturer, the optimal retail price and the retailer’s green advertising effort level, and the optimal profits for each party in the supply chain are respectively given by* $w^{MC}=\frac{\begin{array}{c} P_{c}\left(k\left(z^{\text{2}}+\text{2}v(-\text{1}+\delta )\right)-t\left(P_{c}+t\right)v(-\text{1}+\delta )\right)(-\text{1}+\delta )+\\ a(\text{2}kv{\left(-\text{1}+\delta \right)}^{\text{2}}-P_{c}(P_{c}+t)v{\left(-\text{1}+\delta \right)}^{\text{2}}+kz^{\text{2}}(-\text{1}+\text{2}\delta )) \end{array}}{\text{4}kv{\left(-\text{1}+\delta \right)}^{\text{2}}-{\left(P_{c}+t\right)}^{\text{2}}v{\left(-\text{1}+\delta \right)}^{\text{2}}+kz^{\text{2}}(-\text{2}+\text{3}\delta )}$*,* $e^{MC}=\frac{(a-P_{c})(P_{c}+t)v{\left(-\text{1}+\delta \right)}^{\text{2}}}{\text{4}kv{\left(-\text{1}+\delta \right)}^{\text{2}}-{\left(P_{c}+t\right)}^{\text{2}}v{\left(-\text{1}+\delta \right)}^{\text{2}}+kz^{\text{2}}(-\text{2}+\text{3}\delta )}$*,* $p^{MC}=\frac{\begin{array}{c} P_{c}(k\left(z^{\text{2}}+v(-\text{1}+\delta )\right)-t\left(P_{c}+t\right)v)(-\text{1}+\delta )(-\text{1}+\delta )+\\ a(\text{3}kv{\left(-\text{1}+\delta \right)}^{\text{2}}-P_{c}(P_{c}+t)v{\left(-\text{1}+\delta \right)}^{\text{2}}+kz^{\text{2}}(-\text{1}+\text{2}\delta )) \end{array}}{\text{4}kv{\left(-\text{1}+\delta \right)}^{\text{2}}-{\left(P_{c}+t\right)}^{\text{2}}v{\left(-\text{1}+\delta \right)}^{\text{2}}+kz^{\text{2}}(-\text{2}+\text{3}\delta )}$*,* $\gamma^{MC}=-\frac{k(a-P_{c})z(-\text{1}+\delta )}{\text{4}kv{\left(-\text{1}+\delta \right)}^{\text{2}}-{\left(P_{c}+t\right)}^{\text{2}}v{\left(-\text{1}+\delta \right)}^{\text{2}}+kz^{\text{2}}(-\text{2}+\text{3}\delta )}$*,* $\overset{MC}{\underset{m}{\pi }}(w,e)=\frac{\begin{array}{c} a^{\text{2}}kv(-\text{1}+\delta {)}^{\text{2}}-\text{2}akP_{c}v(-\text{1}+\delta {)}^{\text{2}}+P_{c}(kP_{c}v(-\text{1}+\delta {)}^{\text{2}}+\\ \text{2}E_{g}(\text{4}kv{\left(-\text{1}+\delta \right)}^{\text{2}}-{\left(P_{c}+t\right)}^{\text{2}}v{\left(-\text{1}+\delta \right)}^{\text{2}}+kz^{\text{2}}(-\text{2}+\text{3}\delta ))) \end{array}}{\text{8}kv{\left(-\text{1}+\delta \right)}^{\text{2}}-\text{2}{\left(P_{c}+t\right)}^{\text{2}}v{\left(-\text{1}+\delta \right)}^{\text{2}}+\text{2}kz^{\text{2}}(-\text{2}+\text{3}\delta )}$*, and* $\overset{MC}{\underset{r}{\pi }}(p,\gamma )=\frac{k^{\text{2}}{\left(a-P_{c}\right)}^{\text{2}}v(z^{\text{2}}+\text{2}v(-\text{1}+\delta )){\left(-\text{1}+\delta \right)}^{\text{3}}}{\text{2}{\left(kz^{\text{2}}(\text{2}-\text{3}\delta )-\text{4}kv{\left(-\text{1}+\delta \right)}^{\text{2}}+{\left(P_{c}+t\right)}^{\text{2}}v{\left(-\text{1}+\delta \right)}^{\text{2}}\right)}^{\text{2}}}$.

Building on Lemma 4, further analysis explored the impact of the unit carbon price and initial carbon quotas on carbon reduction, as well as the influence of consumer preferences on supply chain decisions.

**Corollary 4.** *Under the scenario MC, the impact of consumer preferences on supply chain decisions is as follows*:

(*i*) $\frac{\partial e^{MC}}{\partial t}>\text{0}$; $\frac{\partial \overset{MC}{\underset{m}{\pi }}}{\partial t}>\text{0}$; (*ii*) $\frac{\partial \gamma^{MC}}{\partial z}>\text{0}$; $\frac{\partial \overset{MC}{\underset{r}{\pi }}}{\partial z}>\text{0}$*, (a) when* $\delta <\frac{\text{1}}{\text{2}}$ *and* $\text{0}<z\leq \sqrt{\frac{\text{4}v(\text{1}-\delta )(\text{1}-\text{2}\delta )}{\left(\text{2}-\text{3}\delta \right)}}$*; (b)* $\frac{\text{2}}{\text{3}}<\delta <\text{1}$ *and* $z>\sqrt{\frac{\text{4}v(\text{1}-\delta )(\text{1}-\text{2}\delta )}{\left(\text{2}-\text{3}\delta \right)}}$*; (c) when* $\frac{{\left(P_{c}+t\right)}^{\text{2}}v(\text{1}-\delta {)}^{\text{2}}}{z^{\text{2}}(\text{2}-\text{3}\delta )-\text{4}v(\text{1}-\delta )(\text{1}-\text{2}\delta )}>k^{MC}>\frac{{\left(P_{c}+t\right)}^{\text{2}}v(\text{1}-\delta {)}^{\text{2}}}{\text{4}v(\text{1}-\delta {)}^{\text{2}}-z^{\text{2}}(\text{2}-\text{3}\delta )}$*,* $\delta \leq \frac{\text{1}}{\text{2}}$*, and* $z>\sqrt{\frac{\text{4}v(\text{1}-\delta )(\text{1}-\text{2}\delta )}{\left(\text{2}-\text{3}\delta \right)}}$*; (d) when* $\frac{{\left(P_{c}+t\right)}^{\text{2}}v(\text{1}-\delta {)}^{\text{2}}}{z^{\text{2}}(\text{2}-\text{3}\delta )-\text{4}v(\text{1}-\delta )(\text{1}-\text{2}\delta )}>k^{MC}>\frac{{\left(P_{c}+t\right)}^{\text{2}}v(\text{1}-\delta {)}^{\text{2}}}{\text{4}v(\text{1}-\delta {)}^{\text{2}}-z^{\text{2}}(\text{2}-\text{3}\delta )}$ *and* $\frac{\text{1}}{\text{2}}\leq \delta <\frac{\text{2}}{\text{3}}$*.* $\frac{\partial \overset{MC}{\underset{r}{\pi }}}{\partial z}<\text{0}$*, (a) when* $k^{MC}>\frac{{\left(P_{c}+t\right)}^{\text{2}}v(\text{1}-\delta {)}^{\text{2}}}{z^{\text{2}}(\text{2}-\text{3}\delta )-\text{4}v(\text{1}-\delta )(\text{1}-\text{2}\delta )}>\frac{{\left(P_{c}+t\right)}^{\text{2}}v(\text{1}-\delta {)}^{\text{2}}}{\text{4}v(\text{1}-\delta {)}^{\text{2}}-z^{\text{2}}(\text{2}-\text{3}\delta )}$*,* $\delta \leq \frac{\text{1}}{\text{2}}$*, and* $z>\sqrt{\frac{\text{4}v(\text{1}-\delta )(\text{1}-\text{2}\delta )}{\left(\text{2}-\text{3}\delta \right)}}$*; (b) when* $k^{MC}>\frac{{\left(P_{c}+t\right)}^{\text{2}}v(\text{1}-\delta {)}^{\text{2}}}{z^{\text{2}}(\text{2}-\text{3}\delta )-\text{4}v(\text{1}-\delta )(\text{1}-\text{2}\delta )}>\frac{{\left(P_{c}+t\right)}^{\text{2}}v(\text{1}-\delta {)}^{\text{2}}}{\text{4}v(\text{1}-\delta {)}^{\text{2}}-z^{\text{2}}(\text{2}-\text{3}\delta )}$ *and* $\frac{\text{1}}{\text{2}}<\delta \leq \frac{\text{2}}{\text{3}}$*; (c) when* $k^{MC}>\frac{{\left(P_{c}+t\right)}^{\text{2}}v(-\text{1}+\delta {)}^{\text{2}}}{\text{4}v(\text{1}-\delta {)}^{\text{2}}-z^{\text{2}}(\text{2}-\text{3}\delta )}>\frac{{\left(P_{c}+t\right)}^{\text{2}}v(-\text{1}+\delta {)}^{\text{2}}}{z^{\text{2}}(\text{2}-\text{3}\delta )-\text{4}v(\text{1}-\delta )(\text{1}-\text{2}\delta )}$*,* $\frac{\text{2}}{\text{3}}<\delta <\text{1}$*, and* $\text{0}<z\leq \sqrt{\frac{\text{4}v(\text{1}-\delta )(\text{1}-\text{2}\delta )}{\left(\text{2}-\text{3}\delta \right)}}$.

Corollary 4 indicates that when manufacturer shares costs of retailer, manufacturer’s carbon emission reduction efforts, and profits of manufacturer increase with consumer sensitivity to carbon reduction. On one hand, when manufacturer shares retailer’s profits, retailer’s green advertising attracts consumer spending, requiring manufacturer not only to continually improve product’s emission reduction rates but also to share retailer’s costs. Although this can lead to increased benefits from green advertising, excessive cost-sharing ratio can create imbalance, reducing manufacturer’s willingness to produce and cut emissions. Moreover, as retailer’s green advertising efforts increase, attracting more consumers, surge in production volume can lead to decrease in carbon reduction rate for manufacturer.

Corollary 4 also demonstrates that manufacturer sharing retailer’s costs can reduce input costs and increase profits of retailer. When manufacturer shares costs of retailer, retailer intensifies green advertising efforts, stimulating market demand. Manufacturer then increases emission reduction rates to meet market demand, leading to higher emission reduction costs and final selling prices. When manufacturer shares retailer costs and green advertising sensitivity is low, retailer profits increase with sensitivity to green advertising efforts. When emission reduction costs are relatively low, retailer can achieve considerable profits with minimal cost when meeting consumer preferences.

**Corollary 5.** *Under the scenario MC, the impact of the cost-sharing ratio on the decisions of supply chain members is characterized as follows*:

$\frac{\partial \overset{MC}{\underset{m}{\pi }}}{\partial \delta }>\text{0}$*, when* $\delta \leq \frac{\text{1}}{\text{3}}$; $\frac{\partial \overset{MC}{\underset{m}{\pi }}}{\partial \delta }<\text{0}$*, when* $\delta >\frac{\text{1}}{\text{3}}$; $\frac{\partial \overset{MC}{\underset{r}{\pi }}}{\partial \delta }>\text{0}$*, when* ${\frac{{\left(P_{c}+t\right)}^{\text{2}}v(\text{1}-\delta {)}^{\text{2}}}{\text{8}v(\text{1}-\delta )\delta -\text{3}z^{\text{2}}\delta }>k}^{MC}>\frac{{\left(P_{c}+t\right)}^{\text{2}}v(-\text{1}+\delta {)}^{\text{2}}}{\text{4}v(\text{1}-\delta {)}^{\text{2}}-z^{\text{2}}(\text{2}-\text{3}\delta )}$*,* $\delta \leq \frac{\text{1}}{\text{3}}$; $\frac{\partial \overset{MC}{\underset{r}{\pi }}}{\partial \delta }<\text{0}$*, when* $k^{MC}>\frac{{\left(P_{c}+t\right)}^{\text{2}}v(\text{1}-\delta {)}^{\text{2}}}{\text{8}v(\text{1}-\delta )\delta -\text{3}z^{\text{2}}\delta }>\frac{{\left(P_{c}+t\right)}^{\text{2}}v(-\text{1}+\delta {)}^{\text{2}}}{\text{4}v(\text{1}-\delta {)}^{\text{2}}-z^{\text{2}}(\text{2}-\text{3}\delta )}$*,* $\delta \leq \frac{\text{1}}{\text{3}}$ *or* $\delta >\frac{\text{1}}{\text{3}}$*.*

Corollary 5 is proven in the S1 Appendix. It establishes that when manufacturer shares costs with retailer, if the cost-sharing ratio is within one-third, all relevant decision variables increase as the cost-sharing ratio increases. However, when this ratio surpasses the threshold, profits begin to decline.

Corollary 5 suggests that relatively low cost-sharing ratio does not impose significant cost pressures on manufacturer and assists retailer in actively investing in enhancing green advertising efforts, thereby gaining more market share. This also encourages manufacturer to increase carbon reduction rates to meet consumer demands and strengthen product competitiveness. However, if the cost-sharing ratio exceeds the threshold, manufacturer’s profits will decrease.

## 5. Numerical analysis

This section validates the theoretical propositions previously discussed, utilizing numerical simulations to examine changes in optimal decision values for supply chain enterprises. Drawing on prior research [28] and integrating assumptions from this study, the established parameters are as follows: a=50, t=45, z=70, k=5500, v=5500, $p_{c}=\text{15}$, $E_{g}=\text{50}$.

### 5.1. Carbon reduction rate

#### 5.1.1. Impact of carbon trading price on carbon reduction rate.

**Fig 3:**
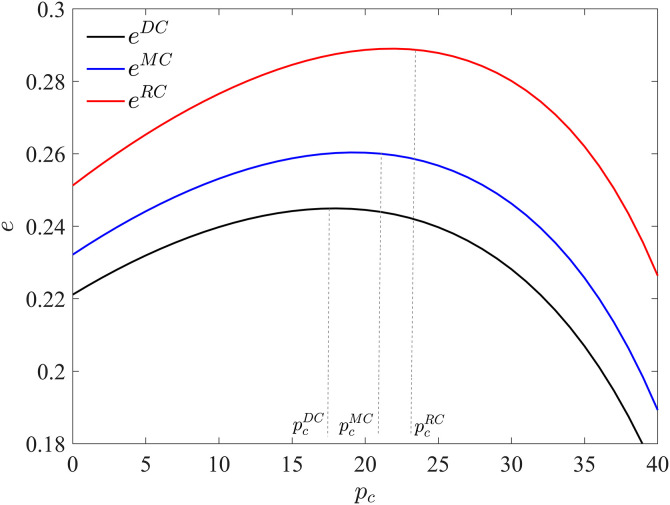
Impact of carbon trading price on carbon reduction rate.

To investigate the impact of carbon trading price on manufacturer carbon reduction rate across different scenarios, we set δ=θ=0.1, with other common parameters remaining unchanged. The trend in carbon reduction rates under three scenarios is illustrated in Fig 3.

Given that $\frac{\partial e^{DC}}{\partial P_{c}}<\text{0}$, when $k^{DC}>\frac{v(t+a)(P_{c}+t{)}^{\text{2}}}{\text{2}(\text{2}P_{c}-a+t)(\text{2}v-z^{\text{2}})}$ and $P_{c}>\frac{a-t}{\text{2}}$, i.e., $P_{c}>\overset{DC}{\underset{c}{P}}$; $\frac{\partial e^{RC}}{\partial P_{c}}<\text{0}$, when $k^{RC}>\frac{v(t+a)(P_{c}+t{)}^{\text{2}}}{\text{2}(\text{2}P_{c}-a+t)(\text{2}v-z^{\text{2}})(\text{1}-\theta )}$, i.e., $P_{c}>\overset{RC}{\underset{c}{P}}$; $\frac{\partial e^{MC}}{\partial P_{c}}<\text{0}$, when $k^{MC}>\frac{(a+t){\left(P_{c}+t\right)}^{\text{2}}v(\text{1}-\delta {)}^{\text{2}}}{\left(\text{2}P_{c}+t-a\right)\left(\text{4}v(\text{1}-\delta {)}^{\text{2}}-z^{\text{2}}(\text{2}-\text{3}\delta )\right)}$, i.e., $P_{c}>\overset{MC}{\underset{c}{P}}$; $\overset{RC}{\underset{c}{P}}>\overset{MC}{\underset{c}{P}}>\overset{DC}{\underset{c}{P}}$. It is observed that in all three scenarios, the manufacturer’s carbon reduction rate initially increases and then decreases with an increase in carbon trading price, consistent with the Corollary 1, 2 and 4.

The results from Fig 3 reveal a non-monotonic effect of carbon trading prices on emission reduction. When carbon prices are relatively low, manufacturers have strong incentives to increase emission reduction in order to generate surplus carbon allowances for trading. However, as carbon prices become sufficiently high, the marginal cost of further emission reduction outweighs its benefits. As a result, firms shift their strategy from reducing emissions to adjusting pricing decisions, leading to reduced investment in emission reduction and lower market demand. This finding highlight that excessively high carbon prices may distort firms’ incentives and weaken emission reduction efforts. Therefore, policymakers should avoid overly aggressive carbon pricing and instead aim for a moderate level that balances environmental objectives with firms’ economic incentives.

#### 5.1.2. Impact of consumer low-carbon preference on carbon reduction rate.

**Fig 4:**
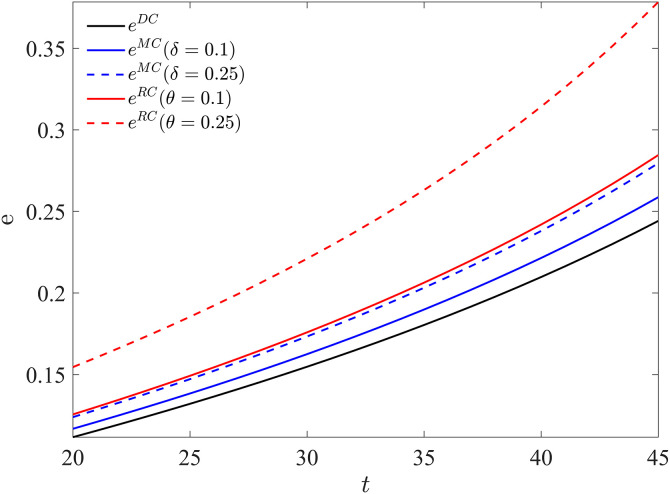
Impact of consumer low-carbon preferences on carbon reduction rate.

To explore the impact of varying cost-sharing ratios on the carbon reduction rate of the manufacturer under three scenarios, we set δ at 0.1 and 0.25, and similarly, θ at 0.1 and 0.25, with other common parameters remaining unchanged. The trends in carbon reduction rates under different cost-sharing models are illustrated in Fig 4.

Given that $\frac{\partial e^{DC}}{\partial t}>\text{0}$, $\frac{\partial e^{RC}}{\partial t}>\text{0}$, and $\frac{\partial e^{MC}}{\partial t}>\text{0}$, the carbon reduction rate of the manufacturer increases with rising consumer sensitivity to emission reduction across all three scenarios. In the *RC* scenario, since $\theta =\text{0}.\text{2}\text{5}<\theta^{h}$, where the threshold $\theta^{h}=\text{1}-\frac{{\left(P_{c}+t\right)}^{\text{2}}v}{\text{4}kv-\text{2}kz^{\text{2}}}$, the carbon reduction rate similarly increases with rising emission sensitivity, consistent with the findings in Corollary 3. In the *MC* scenario, when the cost-sharing ratio within $\delta \leq \frac{\text{1}}{\text{3}}$, the manufacturer’s carbon reduction rate increases with an increase in δ, aligning with the Corollary 5.

Results from Fig 4 show that the consumer’s sensitivity to emissions significantly impacts the variability of the manufacturer’s carbon reduction rate. Within a moderate range, cost-sharing can enhance emission reduction by stimulating green advertising and expanding market demand. However, when the sharing ratio becomes excessively high, it may crowd out the manufacturer’s investment in emission reduction due to increased financial burden, thereby reducing the reduction rate. Similarly, in the scenario where the retailer shares the manufacturer’s costs, a high sharing ratio can impact the retailer’s profits. Moreover, in the retailer’s cost-sharing scenario, the reduction rate is substantially higher than in the other two scenarios, making it the optimal strategy for enhancing the reduction rate.

#### 5.1.3. Impact of consumer low-carbon preferences on manufacturer profit.

**Fig 5:**
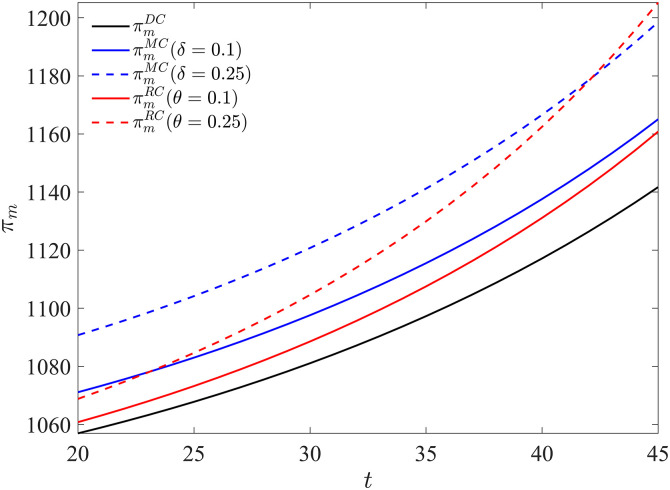
Impact of consumer low-carbon preferences on manufacturer profit.

In analyzing the factors that influence manufacturer profit, we focus on the consumer sensitivity to emission reduction and the cost-sharing ratios in different scenarios. The effects of these variables on manufacturer profit are illustrated in Fig 5.

Given that $\frac{\partial \overset{DC}{\underset{m}{\pi }}}{\partial t}>\text{0}$, $\frac{\partial \overset{RC}{\underset{m}{\pi }}}{\partial t}>\text{0}$, and $\frac{\partial \overset{MC}{\underset{m}{\pi }}}{\partial t}>\text{0}$, manufacturer profit increases with rising consumer sensitivity to emission reduction across all three scenarios. Moreover, in the *RC* scenario, when $\theta <\theta^{h}$, where the threshold $\theta^{h}=\text{1}-\frac{{\left(P_{c}+t\right)}^{\text{2}}v}{\text{4}kv-\text{2}kz^{\text{2}}}$, manufacturer profit increases with the rising cost proportion of carbon reduction borne by the retailer, aligning with the conclusions of Corollary 3. Similarly, in the *MC* scenario, if the cost-sharing ratio continually increases within a range of $\delta \leq \frac{\text{1}}{\text{3}}$, manufacturer profit also increases, consistent with Corollary 5.

Results from Fig 5 indicate a positive correlation between manufacturer profit and consumer sensitivity to emission reduction. When consumer sensitivity is relatively low, the manufacturer achieves higher profit under the model where it shares the retailer’s costs, as increased advertising efforts can still stimulate demand. However, when the manufacturer shares the retailer’s costs, the retailer’s aggressive green advertising efforts, despite the manufacturer incurring higher costs, result in benefits far outweighing the costs, leading to greater manufacturer profit. Therefore, in studying how to maximize manufacturer profit, consideration must be given not only to the cost-sharing ratio but also to consumer sensitivity.

### 5.2. Green advertising efforts

#### 5.2.1. Impact of advertising effectiveness on green advertising efforts.

**Fig 6:**
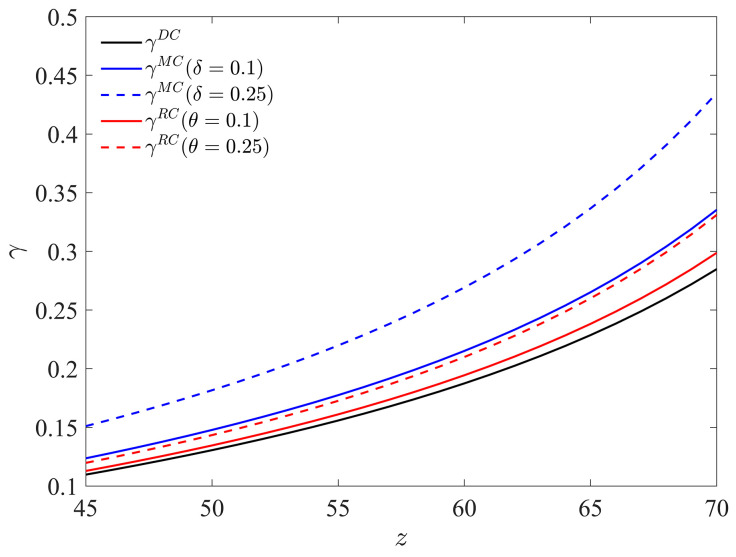
Impact of advertising effectiveness on green advertising efforts.

Addressing the issue of green advertising, we compared the impact of various cost-sharing ratios on retailer green advertising efforts across three scenarios. Under constant common parameters, the relationship between green advertising efforts and cost-sharing ratios was investigated, as shown in Fig 6.

Since $\frac{\partial \gamma^{DC}}{\partial z}>\text{0}$, $\frac{\partial \gamma^{RC}}{\partial z}>\text{0}$, and $\frac{\partial \gamma^{MC}}{\partial z}>\text{0}$, retailer green advertising efforts increase with rising consumer sensitivity to green advertising in all three scenarios. in the *RC* scenario, when $\theta <\theta^{h}$, where the threshold $\theta^{h}=\text{1}-\frac{{\left(P_{c}+t\right)}^{\text{2}}v}{\text{4}kv-\text{2}kz^{\text{2}}}$ is defined, the optimal green advertising efforts also increase with an increase in θ, aligning with the conclusions of Corollary 3. Similarly, In the *MC* scenario, with $\delta =\text{0}.\text{2}\text{5}<\text{1}-\sqrt{\frac{z^{\text{2}}}{\text{4}v}}$, retailer green advertising efforts increase with an increase in δ, consistent with Corollary 5.

Results from Fig 6 indicate that the higher the consumer sensitivity to green advertising, the greater the retailer’s green advertising efforts. Similarly, excessively high cost-sharing ratios may weaken these incentives in both sharing models due to increased financial burden. Moreover, scenarios where the manufacturer shares retailer costs are more favorable for increasing retailer green advertising efforts, as it better aligns incentives for demand expansion. Therefore, if the goal is to attract more consumers through advertising, this decision-making strategy should be considered preferentially.

#### 5.2.2. Impact of advertising effectiveness on retailer profit.

**Fig 7:**
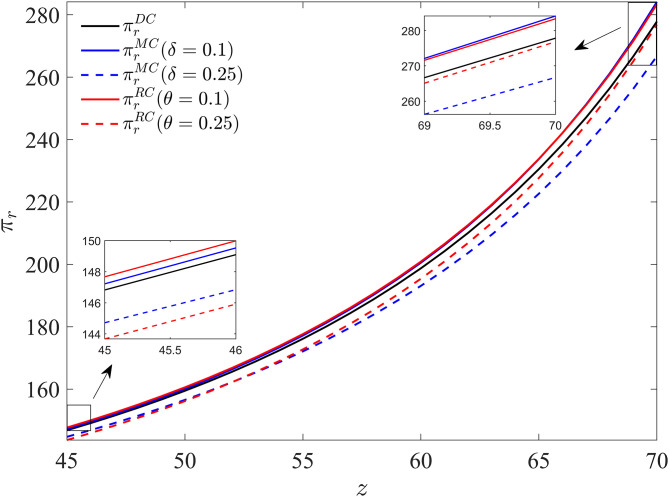
Impact of advertising effectiveness on retailer profit.

To analyze variations in retailer profit, we studied the effects of green advertising effectiveness and the cost-sharing ratios on retailer profit. The trend of retailer profit influenced by these factors is illustrated in Fig 7.

Given that $\delta <\frac{\text{1}}{\text{2}}$ and $\text{0}<z\leq \sqrt{\frac{\text{4}v(\text{1}-\delta )(\text{1}-\text{2}\delta )}{\left(\text{2}-\text{3}\delta \right)}}$, and $\theta \leq \frac{\text{1}}{\text{3}}$ and $k>\frac{{\left(P_{c}+t\right)}^{\text{2}}v}{\text{2}\left(\text{2}v-z^{\text{2}}\right)(\text{1}-\theta )}>\frac{{\left(P_{c}+t\right)}^{\text{2}}v(\text{5}\theta -\text{1})}{\text{2}\left(\text{2}v-z^{\text{2}}\right)(\text{1}-\theta {)}^{\text{2}}}$, $\frac{\partial \overset{DC}{\underset{r}{\pi }}}{\partial z}>\text{0}$, $\frac{\partial \overset{RC}{\underset{r}{\pi }}}{\partial z}>\text{0}$, and $\frac{\partial \overset{MC}{\underset{r}{\pi }}}{\partial z}>\text{0}$, retailer profit increases with the effectiveness of advertising across all three scenarios. Furthermore, in the *RC* scenario, under conditions where $k>\frac{{\left(P_{c}+t\right)}^{\text{2}}v}{\text{4}\left(\text{2}v-z^{\text{2}}\right)\theta }>\frac{{\left(P_{c}+t\right)}^{\text{2}}v}{\text{2}\left(\text{2}v-z^{\text{2}}\right)(\text{1}-\theta )}$, retailer profit also decreases with an increase in θ, aligning with the conclusions of Corollary 3. Similarly, in the *MC* scenario, if $\delta \leq \frac{\text{1}}{\text{3}}$ and $k>\frac{{\left(P_{c}+t\right)}^{\text{2}}v(\text{1}-\delta {)}^{\text{2}}}{\text{8}v(\text{1}-\delta )\delta -\text{3}z^{\text{2}}\delta }>\frac{{\left(P_{c}+t\right)}^{\text{2}}v(-\text{1}+\delta {)}^{\text{2}}}{\text{4}v(\text{1}-\delta {)}^{\text{2}}-z^{\text{2}}(\text{2}-\text{3}\delta )}$, retailer profit decreases with an increase in δ, consistent with the conclusions of Corollary 5.

Results from Fig 7 show that retailer profit positively correlates with consumer sensitivity to green advertising. Likewise, when consumer sensitivity to green advertising is low, retailer profit is higher under the model where the retailer shares the manufacturer’s costs, and vice versa. Moreover, when the manufacturer shares the retailer’s costs, increasing the sharing ratio does not necessarily improve retailer profit, as excessive cost burdens may reduce the manufacturer’s emission reduction efforts and indirectly weaken market demand. Therefore, in maximizing retailer profit, considerations should include not only consumer sensitivity but also the sharing ratio and the manufacturer’s emission reduction costs.

### 5.3. Comparative analysis under different models

#### 5.3.1. Comparison of manufacturer profit.

**Fig 8:**
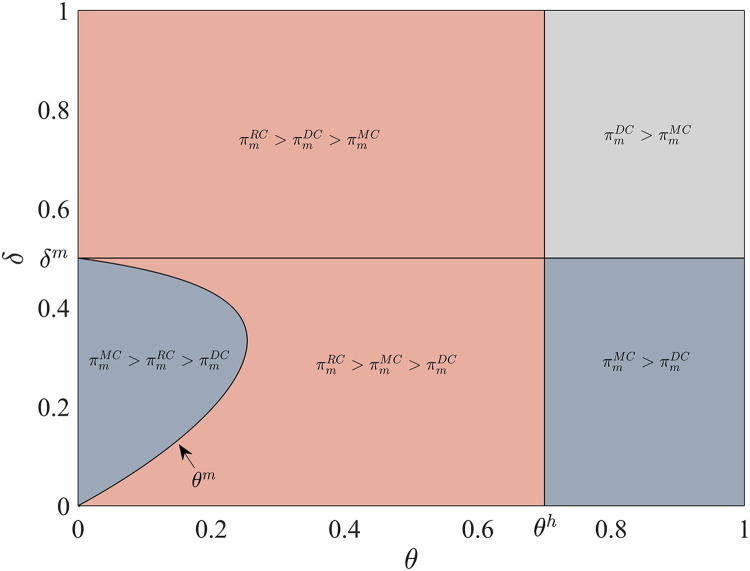
Comparison of manufacturer profit.

The profit of the manufacturer is jointly influenced by the sharing ratio of carbon reduction costs borne by the retailer and the sharing ratio of green advertising costs borne by the manufacturer. A comparative analysis of the manufacturer’s profit under different cost-sharing ratio combinations across various models is presented in Proposition 1, as illustrated in Fig 8 below.

**Proposition 1.** *When* $\theta <\theta^{h}$: (1) *if* $\delta >\delta^{m}$*, then* $\overset{RC}{\underset{m}{\pi }}>\overset{DC}{\underset{m}{\pi }}>\overset{MC}{\underset{m}{\pi }}$; (2) *if* $\delta <\delta^{m}$ *and* $\theta <\theta^{m}$*, then* $\overset{MC}{\underset{m}{\pi }}>\overset{RC}{\underset{m}{\pi }}>\overset{DC}{\underset{m}{\pi }}$; (3) *if* $\delta <\delta^{m}$ *and* $\theta >\theta^{m}$*, then* $\overset{RC}{\underset{m}{\pi }}>\overset{MC}{\underset{m}{\pi }}>\overset{DC}{\underset{m}{\pi }}$*. Otherwise, when* $\theta >\theta^{h}$: (1) *if* $\delta >\delta^{m}$*, then* $\overset{DC}{\underset{m}{\pi }}>\overset{MC}{\underset{m}{\pi }}$; (2) *if* $\delta <\delta^{m}$*, then* $\overset{MC}{\underset{m}{\pi }}>\overset{DC}{\underset{m}{\pi }}$.

Results from Fig 8 indicate that in both cost-sharing models, manufacturer profit is superior to that in the decentralized decision-making model. The manufacturer’s profit advantage is mainly determined by the emission reduction cost coefficient and the carbon price level. When the manufacturer’s emission reduction cost is relatively high or the carbon price is at a moderate level, the *RC* model becomes more favorable, as the retailer’s cost-sharing effectively alleviates the manufacturer’s financial burden and directly motivates greater abatement efforts, thereby enhancing overall profitability. In contrast, the *MC* model improves the manufacturer’s profit in a more indirect way, primarily through the retailer’s intensified green advertising that expands market demand rather than directly reducing production costs. Although the manufacturer may achieve slightly higher profits under the *MC* model when both cost-sharing ratios are relatively low, the *RC* model generally delivers more stable and substantial profit gains due to its direct improvement in cost efficiency. Moreover, within the feasible range defined by the Hessian matrix conditions, the *RC* model not only helps the manufacturer share emission reduction costs but also attracts more consumers through higher abatement levels, thereby further strengthening profitability and improving overall performance.

#### 5.3.2. Comparison of retailer profit.

**Fig 9:**
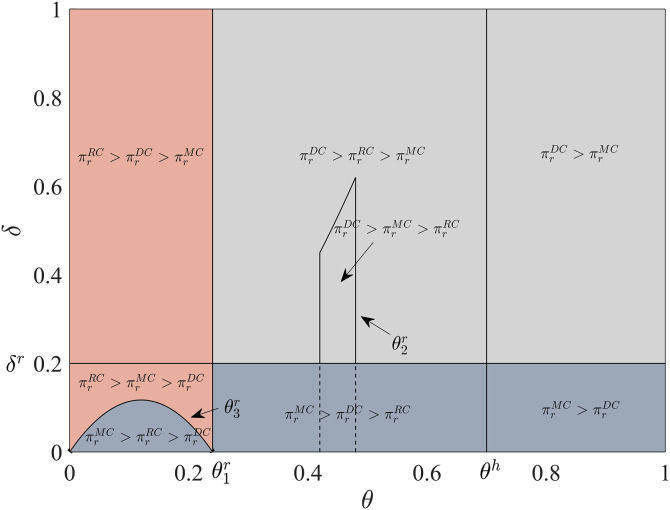
Comparison of retailer profit.

The profit of the retailer is jointly influenced by the sharing ratio of carbon reduction costs borne by the retailer and the sharing ratio of green advertising costs borne by the manufacturer. A comparative analysis of the retailer’s profit under different cost-sharing ratio combinations across various models is presented in Proposition 2, as illustrated in Fig 9 below.

**Proposition 2.** *When* $\theta <\overset{r}{\underset{\text{1}}{\theta }}$*:* (1) *if* $\delta >\delta^{r}$*, then* $\overset{RC}{\underset{r}{\pi }}>\overset{DC}{\underset{r}{\pi }}>\overset{MC}{\underset{r}{\pi }}$; (2) *if* $\delta <\delta^{r}$ *and* $\theta <\overset{r}{\underset{\text{3}}{\theta }}$*, then* $\overset{MC}{\underset{r}{\pi }}>\overset{RC}{\underset{r}{\pi }}>\overset{DC}{\underset{r}{\pi }}$; (3) *if* $\delta <\delta_{\pi_{r}}$ *and* $\theta >\overset{r}{\underset{\text{3}}{\theta }}$*, then* $\overset{RC}{\underset{r}{\pi }}>\overset{MC}{\underset{r}{\pi }}>\overset{DC}{\underset{r}{\pi }}$*. When* $\overset{r}{\underset{\text{1}}{\theta }}<\theta <\theta^{h}$*:* (1) *if* $\delta >\delta^{r}$ *and* $\theta <\overset{r}{\underset{\text{2}}{\theta }}$*, then* $\overset{DC}{\underset{r}{\pi }}>\overset{MC}{\underset{r}{\pi }}>\overset{RC}{\underset{r}{\pi }}$; (2) *if* $\delta >\delta^{r}$ *and* $\theta >\overset{r}{\underset{\text{2}}{\theta }}$*, then* $\overset{DC}{\underset{r}{\pi }}>\overset{RC}{\underset{r}{\pi }}>\overset{MC}{\underset{r}{\pi }}$; (3) *if* $\delta <\delta^{r}$*, then* $\overset{MC}{\underset{r}{\pi }}>\overset{DC}{\underset{r}{\pi }}>\overset{RC}{\underset{r}{\pi }}$*. Otherwise, when* $\theta >\theta^{h}$*:* (1) *if* $\delta >\delta^{r}$*, then* $\overset{DC}{\underset{r}{\pi }}>\overset{MC}{\underset{r}{\pi }}$; (2) *if* $\delta <\delta^{r}$*, then* $\overset{MC}{\underset{r}{\pi }}>\overset{DC}{\underset{r}{\pi }}$*.*

Results from Fig 9 indicate that although the *MC* model can significantly enhance the retailer’s profit, it requires the manufacturer to bear a certain proportion of green advertising costs, which may weaken its emission reduction efforts. This could lead to insufficient investment in abatement and adversely affect consumers who are sensitive to low-carbon products. The retailer’s profit is mainly influenced by the green advertising cost coefficient and consumers’ sensitivity to advertising. When green advertising costs are high or consumers respond strongly to advertising efforts, the *MC* model becomes more advantageous, as the manufacturer’s cost-sharing encourages the retailer to invest more in green advertising and expand market demand. However, when consumers place greater emphasis on the product’s low-carbon attributes, the *RC* model becomes more favorable, as the manufacturer’s stronger abatement efforts indirectly enhance product attractiveness and improve the retailer’s profit. Overall, the *MC* model performs better in stimulating market expansion, whereas the *RC* model is more effective in strengthening the product’s green value.

#### 5.3.3. Comparison of supply chain profit.

**Fig 10:**
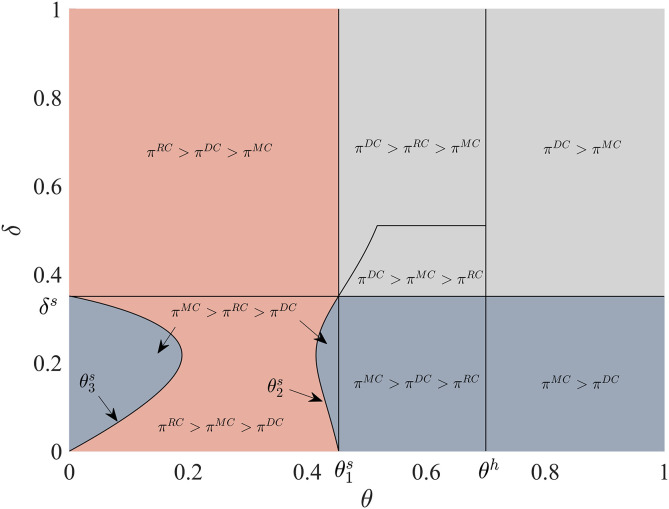
Comparison of supply chain profit (${𝐏}_{c}=15$).

**Fig 11:**
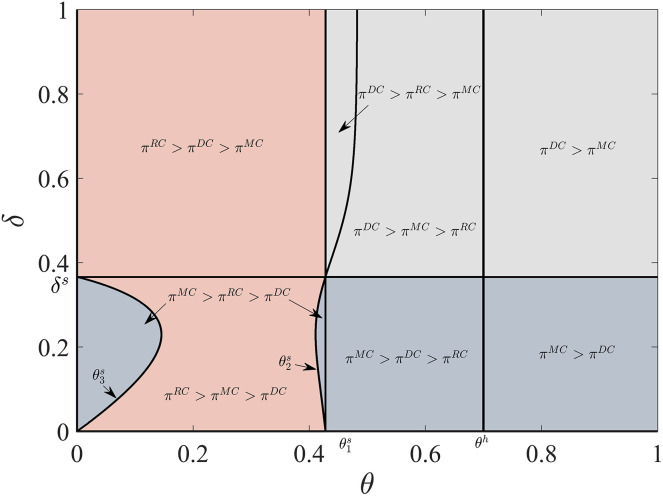
Comparison of supply chain profit (${𝐏}_{c}=25$).

Based on the above analysis, the profit of the supply chain is jointly determined by the sharing ratio of carbon reduction costs borne by the retailer and the sharing ratio of green advertising costs borne by the manufacturer. To further examine how these relationships evolve under different carbon pricing environments, we extend the numerical analysis to consider multiple carbon price scenarios. A comparative analysis of the supply chain’s profit under different cost-sharing ratio combinations across various models is provided in Proposition 3, as depicted in Figs 10 and 11 below.

**Proposition 3.** *When* $\theta <\overset{s}{\underset{\text{1}}{\theta }}$*:* (1) *if* $\delta >\delta^{s}$*, then* $\pi^{RC}>\pi^{DC}>\pi^{MC}$; (2) *if* $\delta <\delta^{s}$ *and* $\theta <\overset{s}{\underset{\text{3}}{\theta }}$*, then* $\pi^{MC}>\pi^{RC}>\pi^{DC}$; (3) *if* $\delta <\delta^{s}$ *and* $\theta >\overset{s}{\underset{\text{3}}{\theta }}$*, then* $\pi^{RC}>\pi^{MC}>\pi^{DC}$*. When* $\overset{s}{\underset{\text{1}}{\theta }}<\theta <\theta^{h}$*:* (1) *if* $\delta >\delta^{s}$ *and* $\theta <\overset{s}{\underset{\text{2}}{\theta }}$*, then* $\pi^{DC}>\pi^{MC}>\pi^{RC}$; (2) *if* $\delta >\delta^{s}$ *and* $\theta >\overset{s}{\underset{\text{2}}{\theta }}$*, then* $\pi^{DC}>\pi^{RC}>\pi^{MC}$; (3) *if* $\delta <\delta^{s}$*, then* $\pi^{MC}>\pi^{DC}>\pi^{RC}$*. Otherwise, when* $\theta >\theta^{h}$*:* (1) *if* $\delta >\delta^{s}$*, then* $\pi^{DC}>\pi^{MC}$; (2) *if* $\delta <\delta^{s}$*, then* $\pi^{MC}>\pi^{DC}$*.*

Results from Figs 10 and 11 indicate: Based on the region diagram for maximizing manufacturer profit, when the retailer shares the manufacturer’s emission reduction cost, an appropriate cost-sharing ratio can enhance the manufacturer’s profit; however, an excessively high ratio may reduce the overall efficiency of the supply chain. For both models under relatively low sharing ratios, it is necessary to further analyze the specific proportions to determine the conditions under which the total supply chain profit is maximized. The overall supply chain profit reflects a trade-off between emission reduction efficiency and market expansion. When consumers exhibit strong low-carbon preferences and the carbon price is relatively high, the *RC* model can maximize the total profit of the supply chain, as the manufacturer’s abatement efforts not only improve social welfare but also stimulate market demand. In contrast, when consumers are more sensitive to advertising than to emission reduction, the *MC* model performs better because increased advertising investment more effectively drives sales. Overall, the optimal outcome for the supply chain depends on how market characteristics and policy factors (such as the carbon price) jointly balance the benefits of emission reduction and advertising. Importantly, comparing the results under different carbon price levels shows that the overall pattern of model selection remains largely unchanged, indicating that the main insights are robust to variations in carbon pricing.

## 6. Conclusion

With escalating concerns over climate issues and heightened consumer awareness of sustainability, the cap-and-trade policy has been broadly implemented to mitigate carbon emissions. This study examines how different supply chain structures, including centralized, decentralized, and partially centralized models, affect manufacturers’ carbon reduction decisions and retailers’ green advertising strategies.

The major findings of this study reveal several important insights. The analysis yields several important insights. First, managers should not rely solely on increasing carbon prices to drive emission reduction. Instead, they need to jointly consider carbon price levels and abatement costs, as excessively high carbon prices may reduce firms’ incentives when reduction costs are substantial. This suggests that a balanced regulatory and operational strategy is necessary. Second, firms should adopt an integrated approach to sustainability investments by coordinating carbon reduction and green advertising decisions. In markets characterized by strong consumer environmental awareness and effective advertising channels, aligning these two strategies can significantly enhance demand and improve overall performance. Third, rather than focusing only on increasing cost-sharing ratios, managers should evaluate the structure of cost-sharing arrangements based on their operational environment. In practice, firms need to assess whether their competitive advantage lies more in production-side improvements (e.g., carbon reduction) or demand-side expansion (e.g., advertising effectiveness), and then choose a coordination mechanism that best aligns incentives across supply chain members. This perspective provides a practical guideline for adapting coordination strategies to different market conditions.

Based on the study’s findings, we offer the following managerial insights: First, managers should carefully evaluate carbon price levels and their interaction with carbon reduction costs. While increasing carbon prices can generally stimulate emission reduction, excessively high prices combined with high abatement costs may lead to diminishing incentives, suggesting that firms should adopt a balanced approach rather than relying solely on price signals. Second, firms should align green advertising strategies with actual carbon reduction efforts, particularly in markets with strong consumer environmental awareness and high advertising effectiveness. In such settings, coordinated investments in both emission reduction and advertising can significantly enhance market performance. Third, the selection of supply chain coordination mechanisms should be contingent on the structure of cost-sharing. Specifically, the RC model is more suitable when carbon reduction cost-sharing is low and advertising cost-sharing is high. When carbon reduction cost-sharing is relatively high, firms should adopt the MC model if advertising cost-sharing is low, whereas the DC model becomes more effective when advertising cost-sharing is high. These results provide clear guidance for managers in choosing appropriate coordination strategies under different operational conditions.

This study identifies several limitations that suggest promising directions for future research. First, the coordination of the partially centralized supply chain in this paper primarily relies on cost-sharing contracts (i.e., RC and MC mechanisms), although other governance approaches such as carbon finance or equity holding are also widely observed in practice. Moreover, these cost-sharing mechanisms may face several implementation challenges. For instance, information asymmetry regarding effort levels (e.g., carbon reduction or advertising investment) may lead to free-riding behavior. In addition, incentive misalignment may arise because the party sharing the cost does not fully control the corresponding decision variable. Contract design and coordination complexity may also increase the difficulty of implementing such mechanisms effectively. Future research could explore alternative coordination schemes or hybrid mechanisms to address these issues. Second, this model focuses solely on the bilateral relationship between a single manufacturer and a single retailer, excluding competitive factors among multiple manufacturers or retailers. Future research may extend the model to scenarios involving multiple manufacturers, multiple retailers, or platform competition, thereby capturing more complex market structures. Lastly, while this study primarily emphasizes economic decision-making, the environmental goals of green sustainable development represent another important research direction. Future studies could further incorporate broader environmental and social objectives, such as welfare implications or long-term sustainability considerations, into the analytical framework.

## Supporting information

S1 Appendix Mathematical proofs. Proofs of Lemmas 1–4, Corollaries 1–5, and Comparative analysis. (DOCX)

S1 File Simulation code. (ZIP)
