# Supplementary material for: Strategies for carbon reduction and advertising investments in partially centralized supply chains
Source: PLoS One. 2026 Jun 16;21(6):e0351412. doi: 10.1371/journal.pone.0351412 (PMC13271493; doi:10.1371/journal.pone.0351412)
Supplement: S1 Appendix — (DOCX) [file pone.0351412.s001.docx]

# SI Appendix. Mathematical proofs.

Proofs of Lemmas 1-4, Corollaries 1-5, and Comparative analysis.

*Proof of Lemma 1*

Under centralized decision-making, the overall profit function for the supply chain is represented as $\pi_{s}(p,e,\gamma)=pD-\frac{ke^{2}}{2}-\frac{v\gamma^{2}}{2}-P_{c}((1-e)D-E_{g})$. The second derivatives of the profit function with respect to $p$, $e$, and $\gamma$ yields as follows: $\frac{\partial^{2}\pi_{s}}{\partial p^{2}}=-2<0$; $\frac{\partial^{2}\pi_{s}}{\partial e^{2}}=2P_{c}t-k<0$; $\frac{\partial^{2}\pi_{s}}{\partial\gamma^{2}}=-v$; $\frac{\partial^{2}\pi_{s}}{\partial p\partial e}=\frac{\partial^{2}\pi_{s}}{\partial e\partial p}=t-P_{c}$; $\frac{\partial^{2}\pi_{s}}{\partial p\partial r}=\frac{\partial^{2}\pi_{s}}{\partial r\partial p}=z$; $\frac{\partial^{2}\pi_{s}}{\partial e\partial r}=\frac{\partial^{2}\pi_{s}}{\partial r\partial e}=P_{c}z$.

The corresponding second-order principal minor of the Hessian matrix is: $\left| \begin{aligned} &\frac{\partial^{2}\pi_{s}}{\partial p^{2}}\frac{\partial^{2}\pi_{s}}{\partial p\partial e} \\ &\frac{\partial^{2}\pi_{s}}{\partial e\partial p}\frac{\partial^{2}\pi_{s}}{\partial e^{2}} \end{aligned} \right|=2k-(P_{c}+t)^{2}>0$; The determinant of the third-order Hessian matrix is: $\left| \begin{aligned} &\frac{\partial^{2}\pi_{s}}{\partial p^{2}}\frac{\partial^{2}\pi_{s}}{\partial p\partial e}\frac{\partial^{2}\pi_{s}}{\partial p\partial r} \\ &\frac{\partial^{2}\pi_{s}}{\partial e\partial p}\frac{\partial^{2}\pi_{s}}{\partial e^{2}}\frac{\partial^{2}\pi_{s}}{\partial e\partial r} \\ &\frac{\partial^{2}\pi_{s}}{\partial r\partial p}\frac{\partial^{2}\pi_{s}}{\partial r\partial e}\frac{\partial^{2}\pi_{s}}{\partial r^{2}} \end{aligned} \right|=kz^{2}+v(P_{c}+t)^{2}-2kv<0$.

Given that $\frac{\partial^{2}\pi_{s}}{\partial p^{2}}=-2<0$, the function $\pi_{s}$ is concave in terms of $p$, $e$, and $\gamma$ only when $2k-(P_{c}+t)^{2}>0$ and $kz^{2}+v(P_{c}+t)^{2}-2kv<0$, indicating the existence of optimal levels of carbon reduction rate, retail price, and green advertising effort that maximize the overall supply chain profit. The first-order derivatives of $\pi_{s}(p,e,\gamma)$ with respect to $p$, $e$, and $\gamma$ are calculated and set to zero to find these optimal values:

$$\left\{ \begin{aligned} &\frac{\partial\pi_{s}}{\partial p}=a-2p+z\gamma+P_{c}+e\left( t-P_{c} \right)=0 \\ &\frac{\partial\pi_{s}}{\partial e}=pt-ke-P_{c}\left( t-a+p-2te-z\gamma\right)=0 \\ &\frac{\partial\pi_{s}}{\partial\gamma}=pz-v\gamma-P_{c}z\left( 1-e \right)=0 \end{aligned} \right.$$

Solving the system of equations concurrently provides the equilibrium outcomes for retail price, carbon reduction rate, green advertising effort, and overall supply chain profit, as demonstrated in Lemma 1.

*Proof of Lemma 2*

Under decentralized decision-making, the manufacturer's profit is given by $\pi_{m}^{DC}(w,e)=wD-\frac{ke^{2}}{2}-P_{c}((1-e)D-E_{g})$, while the retailer's profit is $\pi_{r}^{DC}(p,\gamma)=(p-w)D-\frac{v\gamma^{2}}{2}$. In this setup, the manufacturer initially sets the wholesale price $w^{DC}$ and the carbon reduction rate $e^{DC}$. Subsequently, the retailer decides on the retail price $p^{DC}$ and the green advertising effort $\gamma^{DC}$ based on the manufacturer's decisions. Using a backward induction method, the second-order partial derivatives of $\pi_{r}^{DC}$ with respect to $p^{DC}$ and $\gamma^{DC}$ are calculated, and the Hessian matrix is constructed:

$\frac{\partial^{2}\pi_{r}^{DC}}{\partial p^{2}}=-2$; $\frac{\partial^{2}\pi_{r}^{DC}}{\partial\gamma^{2}}=-v$; $\frac{\partial^{2}\pi_{r}^{DC}}{\partial p\partial\gamma}=\frac{\partial^{2}\pi_{r}^{DC}}{\partial\gamma\partial p}=z$. The determinant of the Hessian matrix is $\left| \begin{aligned} &\frac{\partial^{2}\pi_{r}^{DC}}{\partial p^{2}}\frac{\partial^{2}\pi_{r}^{DC}}{\partial p\partial\gamma} \\ &\frac{\partial^{2}\pi_{r}^{DC}}{\partial\gamma\partial p}\frac{\partial^{2}\pi_{r}^{DC}}{\partial\gamma^{2}} \end{aligned} \right|=2v-z^{2}$.

Given that $\frac{\partial^{2}\pi_{r}^{DC}}{\partial p^{2}}=-2<0$, $\pi_{r}^{DC}$ is concave with respect to $p^{DC}$ and $\gamma^{DC}$ when $2v-z^{2}>0$, indicating the existence of optimal retail prices and green advertising efforts that maximize retailer profit. The first-order derivatives are then computed and set to zero:

$$\left\{ \begin{aligned} &\frac{\partial\pi_{r}^{DC}}{\partial p}=a-2p+te+z\gamma+w=0 \\ &\frac{\partial\pi_{r}^{DC}}{\partial\gamma}=pz-v\gamma-wz=0 \end{aligned} \right.$$

Solving the equations concurrently yields the optimal retail price and green advertising level based on the wholesale price and carbon reduction rate. These are then substituted into the manufacturer's profit function $\pi_{m}^{DC}(w,e)=wD-\frac{ke^{2}}{2}-P_{c}((1-e)D-E_{g})$ to derive a profit formula solely dependent on $w$ and $e$. Further analysis involves taking second-order partial derivatives of $\pi_{m}^{DC}$ with respect to $w$ and $e$ and constructing the Hessian matrix:

$\frac{\partial^{2}\pi_{m}^{DC}}{\partial w^{2}}=\frac{2v}{z^{2}-2v}$; $\frac{\partial^{2}\pi_{m}^{DC}}{\partial e^{2}}=\frac{2kv-kz^{2}-2P_{c}vt}{z^{2}-2v}$; $\frac{\partial^{2}\pi_{m}^{DC}}{\partial w\partial e}=\frac{\partial^{2}\pi_{m}^{DC}}{\partial e\partial w}=\frac{P_{c}v-tv}{z^{2}-2v}$. The determinant of this Hessian matrix is $\left| \begin{aligned} &\frac{\partial^{2}\pi_{m}^{DC}}{\partial w^{2}}\frac{\partial^{2}\pi_{m}^{DC}}{\partial w\partial e} \\ &\frac{\partial^{2}\pi_{m}^{DC}}{\partial e\partial w}\frac{\partial^{2}\pi_{m}^{DC}}{\partial e^{2}} \end{aligned} \right|=\frac{2kv(2v-z^{2})-(P_{c}+t)^{2}v^{2}}{(z^{2}-2v)^{2}}$.

Given the assumptions that $2v-z^{2}>0$ and $v>0$, which imply $\frac{\partial^{2}\pi_{m}^{DC}}{\partial w^{2}}=\frac{2v}{z^{2}-2v}<0$, $\pi_{m}^{DC}$ is concave in terms of $w^{DC}$ and $e^{DC}$ when $\frac{2kv(2v-z^{2})-(P_{c}+t)^{2}v^{2}}{(z^{2}-2v)^{2}}>0$, ensuring the existence of optimal wholesale prices and carbon reduction rates that maximize manufacturer profit. First-order derivatives are taken and set to zero:

$$\left\{ \begin{aligned} &\frac{\partial\pi_{m}^{DC}}{\partial w}=\frac{2wv-P_{c}v-va-tev+P_{c}ev}{z^{2}-2v}=0 \\ &\frac{\partial\pi_{m}^{DC}}{\partial e}=\frac{P_{c}vt-wvt-P_{c}av-2P_{c}evt+P_{c}vw-kez^{2}+2kev}{z^{2}-2v}=0 \end{aligned} \right.$$

Solving these equations provides the optimal wholesale prices, carbon reduction rates, retail prices, and green advertising efforts, as well as the optimal profits for both the manufacturer and retailer. The equilibrium outcomes are detailed in Lemma 2.

*Proof of Lemma 3*

Under the scenario where the retailer shares the cost of the manufacturer's low carbon reduction, the manufacturer's profit function is represented as: $\pi_{m}^{RC}\left( w,e \right)=wD-(1-\lambda)\frac{ke^{2}}{2}-P_{c}(\left( 1-e \right)D-E_{g})$. The retailer's profit function is given by: $\pi_{r}^{RC}\left( p,\gamma\right)=\left( p-w \right)D-\frac{v\gamma^{2}}{2}-\lambda\frac{ke^{2}}{2}$. Initially, the manufacturer sets the wholesale price $w^{RC}$ and the carbon reduction rate $e^{RC}$. The retailer then decides the retail price $p^{RC}$ and the green advertising effort $\gamma^{RC}$ based on the manufacturer's decisions.

The optimization begins with the calculation of the second-order partial derivatives of $\pi_{r}^{RC}$ with respect to $p^{RC}$ and $\gamma^{RC}$, leading to the formation of the Hessian matrix:

$\frac{\partial^{2}\pi_{r}^{RC}}{\partial p^{2}}=-2$; $\frac{\partial^{2}\pi_{r}^{RC}}{\partial\gamma^{2}}=-v$; $\frac{\partial^{2}\pi_{r}^{RC}}{\partial p\partial\gamma}=\frac{\partial^{2}\pi_{r}^{RC}}{\partial\gamma\partial p}=z$. The determinant of the Hessian matrix is: $\left| \begin{aligned} &\frac{\partial^{2}\pi_{r}^{RC}}{\partial p^{2}}\frac{\partial^{2}\pi_{r}^{RC}}{\partial p\partial\gamma} \\ &\frac{\partial^{2}\pi_{r}^{RC}}{\partial\gamma\partial p}\frac{\partial^{2}\pi_{r}^{RC}}{\partial\gamma^{2}} \end{aligned} \right|=2v-z^{2}$.

Since $\frac{\partial^{2}\pi_{r}^{RC}}{\partial p^{2}}=-2<0$, $\pi_{r}^{RC}$ is a concave function with respect to $p^{RC}$ and $\gamma^{RC}$ when $2v-z^{2}>0$. This suggests the existence of optimal retail prices and green advertising efforts that maximize the retailer's profit. Setting the first-order derivatives to zero yields the equations:

$$\left\{ \begin{aligned} &\frac{\partial\pi_{r}^{RC}}{\partial p}=a-2p+te+z\gamma+w=0 \\ &\frac{\partial\pi_{r}^{RC}}{\partial\gamma}=pz-wz-v\gamma=0 \end{aligned} \right.$$

Solving this system of equations yields the optimal retail price and green advertising effort in terms of wholesale price and carbon reduction rate. These values are then substituted back into the manufacturer's profit function $\pi_{m}^{RC}$, and second-order partial derivatives with respect to $w$ and $e$ are computed to construct another Hessian matrix:

$\frac{\partial^{2}\pi_{m}^{RC}}{\partial w^{2}}=\frac{2v}{-2v+z^{2}}$; $\frac{\partial^{2}\pi_{m}^{RC}}{\partial e^{2}}=-\frac{2P_{c}tv}{-2v+z^{2}}+k(-1+\theta)$; $\frac{\partial^{2}\pi_{m}^{RC}}{\partial w\partial e}=\frac{\partial^{2}\pi_{m}^{RC}}{\partial e\partial w}=\frac{(P_{c}-t)v}{-2v+z^{2}}$. The determinant of this matrix is: $\left| \begin{aligned} &\frac{\partial^{2}\pi_{m}^{RC}}{\partial w^{2}}\frac{\partial^{2}\pi_{m}^{RC}}{\partial w\partial e} \\ &\frac{\partial^{2}\pi_{m}^{RC}}{\partial e\partial w}\frac{\partial^{2}\pi_{m}^{RC}}{\partial e^{2}} \end{aligned} \right|=-\frac{v({(P_{c}+t)}^{2}v+2k(2v-z^{2})(-1+\theta))}{{(-2v+z^{2})}^{2}}$.

Given the negative $\frac{\partial^{2}\pi_{m}^{RC}}{\partial w^{2}}=\frac{2v}{-2v+z^{2}}<0$ and the positive overall determinant, $\pi_{m}^{RC}$ is concave with respect to $w^{RC}$ and $e^{RC}$. This implies the existence of optimal wholesale pricing and carbon reduction rates that maximize the manufacturer's profit. Solving the corresponding first-order derivatives:

$$\left\{ \begin{aligned} &\frac{\partial\pi_{m}^{RC}}{\partial w}=\frac{v(a+P_{c}-eP_{c}+et-2w)}{2v-z^{2}}=0 \\ &\frac{\partial\pi_{m}^{RC}}{\partial e}=\frac{2eP_{c}tv+v(aP_{c}+tw-P_{c}(t+w))+ek(2v-z^{2})(-1+\theta)}{2v-z^{2}}=0 \end{aligned} \right.$$

Solving these equations delivers the manufacturer's optimal wholesale pricing and carbon reduction rate. This analysis ultimately leads to the determination of optimal retail pricing, green advertising efforts, and the maximization of profits for both the manufacturer and the retailer.

*Proof of Lemma 4*

Under the scenario where the manufacturer shares the retailer's green advertising costs, the manufacturer's profit is given by: $\pi_{m}^{MC}\left( w,e \right)=wD-\frac{ke^{2}}{2}-P_{c}(\left( 1-e \right)D-E_{g})-\delta\frac{v\gamma^{2}}{2}$ and the retailer's profit is: $\pi_{r}^{MC}(p,\gamma)=(p-w)D-(1-\delta)\frac{v\gamma^{2}}{2}$. Initially, the manufacturer determines the wholesale price $w^{MC}$ and the carbon reduction rate $e^{MC}$. Subsequently, the retailer decides the retail price $p^{MC}$ and the green advertising effort $\gamma^{MC}$ based on the manufacturer's decisions. Using the backward induction approach, we first take the second-order partial derivatives of $\pi_{r}^{MC}$ with respect to $p^{MC}$ and $\gamma^{MC}$ and construct the Hessian matrix:

$\frac{\partial^{2}\pi_{r}^{MC}}{\partial p^{2}}=-2$; $\frac{\partial^{2}\pi_{r}^{MC}}{\partial\gamma^{2}}=-v(1-\delta)$; $\frac{\partial^{2}\pi_{r}^{MC}}{\partial p\partial\gamma}=\frac{\partial^{2}\pi_{r}^{MC}}{\partial\gamma\partial p}=z$. The determinant of the Hessian matrix is: $\left| \begin{aligned} &\frac{\partial^{2}\pi_{r}^{MC}}{\partial p^{2}}\frac{\partial^{2}\pi_{r}^{MC}}{\partial p\partial\gamma} \\ &\frac{\partial^{2}\pi_{r}^{MC}}{\partial\gamma\partial p}\frac{\partial^{2}\pi_{r}^{MC}}{\partial\gamma^{2}} \end{aligned} \right|=-z^{2}-2v(-1+\delta)$.

Given that $\frac{\partial^{2}\pi_{r}^{MC}}{\partial p^{2}}=-2<0$, the retailer's profit function $\pi_{r}^{MC}$ is concave with respect to $p^{MC}$ and $\gamma^{MC}$ when $-z^{2}-2v(-1+\delta)>0$. This implies the existence of optimal retail price and green advertising effort that maximize the retailer's profit. Solving the corresponding first-order derivatives:

$$\left\{ \begin{aligned} &\frac{\partial\pi_{r}^{MC}}{\partial p}=a-2p+te+z\gamma+w=0 \\ &\frac{\partial\pi_{r}^{MC}}{\partial\gamma}=pz-wz+v\gamma(-1+\delta)=0 \end{aligned} \right.$$

Solving this system of equations yields the optimal retail price and green advertising effort in terms of wholesale price and carbon reduction rate. Substituting these values back into the manufacturer's profit function $\pi_{m}^{MC}$, the second-order partial derivatives with respect to $w$ and $e$ are taken to form another Hessian matrix:

$\frac{\partial^{2}\pi_{m}^{MC}}{\partial w^{2}}=\frac{v(z^{2}(2-3\delta)-4v{(-1+\delta)}^{2})}{{(z^{2}+2v(-1+\delta))}^{2}}$; $\frac{\partial^{2}\pi_{m}^{MC}}{\partial e^{2}}=-k+\frac{tv(2P_{c}(z^{2}+2v(-1+\delta))(-1+\delta)-tz^{2}\delta)}{{(z^{2}+2v(-1+\delta))}^{2}}$; $\frac{\partial^{2}\pi_{m}^{MC}}{\partial w\partial e}=\frac{\partial^{2}\pi_{m}^{MC}}{\partial e\partial w}=\frac{v(-P_{c}(z^{2}+2v(-1+\delta))(-1+\delta)+t(2v{(-1+\delta)}^{2}+z^{2}(-1+2\delta)))}{{(z^{2}+2v(-1+\delta))}^{2}}$. The determinant of this Hessian matrix is: $\left| \begin{aligned} &\frac{\partial^{2}\pi_{m}^{MC}}{\partial w^{2}}\frac{\partial^{2}\pi_{m}^{MC}}{\partial w\partial e} \\ &\frac{\partial^{2}\pi_{m}^{MC}}{\partial e\partial w}\frac{\partial^{2}\pi_{m}^{MC}}{\partial e^{2}} \end{aligned} \right|=\frac{v(4kv{(-1+\delta)}^{2}-{(P_{c}+t)}^{2}v{(-1+\delta)}^{2}+kz^{2}(-2+3\delta))}{{(z^{2}+2v(-1+\delta))}^{2}}$.

Since $-z^{2}-2v(-1+\delta)>0$, it follows that $\frac{\partial^{2}\pi_{m}^{MC}}{\partial w^{2}}=\frac{v(z^{2}(2-3\delta)-4v{(-1+\delta)}^{2})}{{(z^{2}+2v(-1+\delta))}^{2}}<0$. Moreover, when $v\left( 4kv\left( -1+\delta\right)^{2}-\left( P_{c}+t \right)^{2}v\left( -1+\delta\right)^{2}+kz^{2}\left( -2+3\delta\right) \right)>0$, the manufacturer's profit function $\pi_{m}^{MC}$ is concave in $w^{MC}$ and $e^{MC}$, suggesting the existence of an optimal wholesale price and carbon reduction rate that maximize the manufacturer's profit. Solving the corresponding first-order derivatives:

$$\left\{ \begin{aligned} &\frac{\partial\pi_{m}^{MC}}{\partial w}=\frac{\begin{aligned} v((a+P_{c}-eP_{c}+et-2w)(2v-z^{2})-4v(a+P_{c}-eP_{c}+et-2w)\delta+ \\ (2a+P_{c}-eP_{c}+2et-3w)z^{2}\delta+2v(a+P_{c}-eP_{c}+et-2w)\delta^{2}) \end{aligned}}{{(z^{2}+2v(-1+\delta))}^{2}}=0 \\ &\frac{\partial\pi_{m}^{MC}}{\partial e}=\frac{\begin{aligned} -e\left( k\left( z^{2}+2v\left( -1+\delta\right) \right)^{2}+tv\left( -2P_{c}\left( z^{2}+2v\left( -1+\delta\right) \right)\left( -1+\delta\right)+tz^{2}\delta\right) \right)+ \\ v(-P_{c}(t+w)(z^{2}+2v(-1+\delta))(-1+\delta)+ \\ a(P_{c}(z^{2}+2v(-1+\delta))(-1+\delta)-tz^{2}\delta)+tw(2v{(-1+\delta)}^{2}+z^{2}(-1+2\delta))) \end{aligned}}{{(z^{2}+2v(-1+\delta))}^{2}}=0 \end{aligned} \right.$$

These equations are solved to determine the optimal wholesale price and carbon reduction rate for the manufacturer. The final outcomes yield optimal retail pricing, green advertising efforts, and maximum profits for both the manufacturer and retailer.

*Proof of Corollary 1*

In the fully decentralized scenario, the impact of consumer low-carbon preferences on equilibrium outcomes is explored. With the rise in consumer low-carbon preferences, carbon reduction rates, green advertising efforts, manufacturer's and retailer's profits all increase, expressed by $\frac{\partial e^{DC}}{\partial t}=\frac{\left( a-P_{c} \right)v\left( \left( 4k+\left( P_{c}+t \right)^{2} \right)v-2kz^{2} \right)}{\left( \left( P_{c}+t \right)^{2}v+2k\left( -2v+z^{2} \right) \right)^{2}}>0$, $\frac{\partial\pi_{m}^{DC}}{\partial t}=\frac{k\left( a-P_{c} \right)^{2}\left( P_{c}+t \right)v^{2}}{\left( \left( P_{c}+t \right)^{2}v+2k\left( -2v+z^{2} \right) \right)^{2}}>0$.

*Proof of Corollary 2*

In the scenario where the retailer shares the cost of carbon reduction with the manufacturer (*RC* model), the effects of consumer low-carbon preferences and green advertising efforts on carbon reduction rates, green advertising efforts, manufacturer profits, and retailer profits are analyzed as follows. An increase in consumer low-carbon preferences leads to higher carbon reduction rates, green advertising efforts, and manufacturer profits, expressed as $\frac{\partial e^{RC}}{\partial t}=\frac{\left( a-P_{c} \right)v\left( \left( P_{c}+t \right)^{2}v+2k\left( 2v-z^{2} \right)\left( 1-\theta\right) \right)}{\left( \left( P_{c}+t \right)^{2}v+2k\left( 2v-z^{2} \right)\left( -1+\theta\right) \right)^{2}}>0$, and $\frac{\partial\pi_{m}^{RC}}{\partial t}=\frac{k\left( a-P_{c} \right)^{2}\left( P_{c}+t \right)v^{2}\left( 1-\theta\right)}{\left( \left( P_{c}+t \right)^{2}v-2k\left( 2v-z^{2} \right)\left( 1-\theta\right) \right)^{2}}>0$ respectively.

The enhancement of advertising effectiveness positively influences carbon reduction rates, green advertising efforts, and manufacturer profits, denoted by $\frac{\partial\gamma^{RC}}{\partial z}=\frac{k\left( a-P_{c} \right)\left( 2k\left( 2v+z^{2} \right)\left( 1-\theta\right)-\left( P_{c}+t \right)^{2}v \right)\left( 1-\theta\right)}{\left( \left( P_{c}+t \right)^{2}v+2k\left( 2v-z^{2} \right)\left( -1+\theta\right) \right)^{2}}>0$. The effect of increased advertising effectiveness on retailer profits, $\frac{\partial\pi_{r}^{RC}}{\partial z}=\frac{k^{2}\left( a-P_{c} \right)^{2}vz\left( \left( P_{c}+t \right)^{2}v\left( 1-5\theta\right)+2k\left( 2v-z^{2} \right)\left( 1-\theta\right)^{2} \right)\left( 1-\theta\right)}{\left( 2k\left( 2v-z^{2} \right)\left( 1-\theta\right)-\left( P_{c}+t \right)^{2}v \right)^{3}}$, correlates with the sign of $\left( P_{c}+t \right)^{2}v\left( 1-5\theta\right)+2k\left( 2v-z^{2} \right)\left( 1-\theta\right)^{2}$.

*Proof of Corollary 3*

In the Retailer's Cost-sharing (*RC*) scenario, the impact of the proportion of carbon reduction costs shared by the retailer, denoted as $\theta$, on equilibrium outcomes is analyzed. Under the *RC* model, the manufacturer's profit, $\pi_{m}^{RC}$, in terms of the wholesale price $w^{RC}$ and carbon reduction rate $e^{RC}$, has a Hessian matrix determinant represented as $\left| \begin{aligned} &\frac{\partial^{2}\pi_{m}^{RC}}{\partial w^{2}}\frac{\partial^{2}\pi_{m}^{RC}}{\partial w\partial e} \\ &\frac{\partial^{2}\pi_{m}^{RC}}{\partial e\partial w}\frac{\partial^{2}\pi_{m}^{RC}}{\partial e^{2}} \end{aligned} \right|=-\frac{v({(P_{c}+t)}^{2}v+2k(2v-z^{2})(-1+\theta))}{{(-2v+z^{2})}^{2}}$. This determinant is positive only when $-\frac{v({(\mathrm{pc}+t)}^{2}v+2k(2v-z^{2})(-1+\theta))}{{(-2v+z^{2})}^{2}}>0$, which is true when $\theta<1-\frac{{(\mathrm{pc}+t)}^{2}v}{4kv-2kz^{2}}$. Under these conditions, $\pi_{m}^{RC}$ is a concave function in terms of $w^{RC}$ and $e^{RC}$, indicating the existence of optimal wholesale price and carbon reduction rate that maximize the manufacturer's profit.

When the constraint $\theta^{h}=1-\frac{{(\mathrm{pc}+t)}^{2}v}{4kv-2kz^{2}}$ is met, i.e., $\theta<\theta^{h}$, increases in the proportion of carbon reduction costs shared by the retailer $\theta$, lead to increases in the carbon reduction rate, green advertising efforts, and manufacturer's profits. This is quantitatively supported by the positive derivatives $\frac{\partial e^{RC}}{\partial\theta}=\frac{2k\left( a-P_{c} \right)\left( P_{c}+t \right)v\left( 2v-z^{2} \right)}{\left( \left( P_{c}+t \right)^{2}v+2k\left( 2v-z^{2} \right)\left( -1+\theta\right) \right)^{2}}>0$, $\frac{\partial\gamma^{RC}}{\partial\theta}=\frac{k\left( a-P_{c} \right)\left( P_{c}+t \right)^{2}vz}{\left( \left( P_{c}+t \right)^{2}v+2k\left( 2v-z^{2} \right)\left( -1+\theta\right) \right)^{2}}>0$, and $\frac{\partial\pi_{m}^{RC}}{\partial\theta}=\frac{k\left( a-P_{c} \right)^{2}\left( P_{c}+t \right)^{2}v^{2}}{2\left( \left( P_{c}+t \right)^{2}v+2k\left( 2v-z^{2} \right)\left( -1+\theta\right) \right)^{2}}>0$.

As the proportion of carbon reduction costs shared by the retailer, denoted as $\theta$, increases in the Retailer's Cost-sharing (*RC*) scenario, the impact on the retailer's profits is expressed as $\frac{\partial\pi_{r}^{RC}}{\partial\theta}=\frac{k\left( a-P_{c} \right)^{2}\left( P_{c}+t \right)^{2}v^{2}\left( \left( P_{c}+t \right)^{2}v+4k\left( -2v+z^{2} \right)\theta\right)}{2\left( 2k\left( 2v-z^{2} \right)\left( 1-\theta\right)-\left( P_{c}+t \right)^{2}v \right)^{3}}$. The sign of $\frac{\partial\pi_{r}^{RC}}{\partial\theta}$ corresponds to the sign of $\left( P_{c}+t \right)^{2}v+4k\left( -2v+z^{2} \right)\theta$.

*Proof of Corollary 4*

In the Manufacturer's Cost-sharing (*MC*) scenario for green advertising, the impact of consumer low-carbon preferences and green advertising effectiveness on equilibrium outcomes is analyzed. As consumer low-carbon preferences continually increase, carbon reduction rates, green advertising efforts, manufacturer profits, and retailer profits all increase, denoted by $\frac{\partial e^{MC}}{\partial t}=\frac{\left( a-P_{c} \right)v\left( -1+\delta\right)^{2}\left( 4kv\left( -1+\delta\right)^{2}+\left( P_{c}+t \right)^{2}v\left( -1+\delta\right)^{2}+kz^{2}\left( -2+3\delta\right) \right)}{\left( 4kv\left( -1+\delta\right)^{2}-\left( P_{c}+t \right)^{2}v\left( -1+\delta\right)^{2}+kz^{2}\left( -2+3\delta\right) \right)^{2}}>0$, $\frac{\partial\pi_{m}^{MC}}{\partial t}=\frac{k\left( a-P_{c} \right)^{2}\left( P_{c}+t \right)v^{2}\left( -1+\delta\right)^{4}}{\left( 4kv\left( -1+\delta\right)^{2}-\left( P_{c}+t \right)^{2}v\left( -1+\delta\right)^{2}+kz^{2}\left( -2+3\delta\right) \right)^{2}}>0$.

The following analysis explores the impact of green advertising effectiveness on equilibrium outcomes. As green advertising effectiveness increases, the optimal level of advertising effort also increases, denoted by $\frac{\partial\gamma^{MC}}{\partial z}=\frac{k\left( a-P_{c} \right)\left( 1-\delta\right)(4kv\left( -1+\delta\right)^{2}-\left( P_{c}+t \right)^{2}v\left( -1+\delta\right)^{2}+kz^{2}\left( 2-3\delta\right))}{\left( 4kv\left( -1+\delta\right)^{2}-\left( P_{c}+t \right)^{2}v\left( -1+\delta\right)^{2}+kz^{2}\left( -2+3\delta\right) \right)^{2}}>0$.

The influence of increased green advertising effectiveness on retailer profits is denoted by $\frac{\partial\pi_{r}^{MC}}{\partial z}=-\frac{k^{2}\left( a-P_{c} \right)^{2}vz\left( -1+\delta\right)^{3}\left( \left( P_{c}+t \right)^{2}v\left( 1-\delta\right)^{2}+4kv\left( 1-\delta\right)\left( 1-2\delta\right)-kz^{2}\left( 2-3\delta\right) \right)}{\left( 4kv\left( -1+\delta\right)^{2}-\left( P_{c}+t \right)^{2}v\left( -1+\delta\right)^{2}-kz^{2}\left( 2-3\delta\right) \right)^{3}}$. The sign of this derivative $\frac{\partial\pi_{r}^{MC}}{\partial z}$ is consistent with the expression $\left( P_{c}+t \right)^{2}v\left( -1+\delta\right)^{2}+4kv\left( 1-\delta\right)\left( 1-2\delta\right)-kz^{2}\left( 2-3\delta\right)$.

*Proof of Corollary 5*

In the *MC* scenario model where the manufacturer shares the cost of green advertising, the impact of the sharing ratio $\delta$ on the equilibrium outcome is analyzed as follows. The impact of the manufacturer’s share of green advertising costs $\delta$ on carbon reduction can be expressed as $\frac{\partial e^{MC}}{\partial\delta}=\frac{k\left( a-P_{c} \right)\left( P_{c}+t \right)vz^{2}\left( 1-\delta\right)\left( 1-3\delta\right)}{\left( 4kv\left( -1+\delta\right)^{2}-\left( P_{c}+t \right)^{2}v\left( -1+\delta\right)^{2}+kz^{2}\left( -2+3\delta\right) \right)^{2}}$. It can be seen that the sign of $\frac{\partial e^{MC}}{\partial\delta}$ is related to $\left( 1-3\delta\right)$. When $\delta\leq\frac{1}{3}$, $\frac{\partial e^{MC}}{\partial\delta}>0$, indicating that the manufacturer’s carbon reduction rate increases as the share of green advertising costs increases. When $\delta>\frac{1}{3}$, $\frac{\partial e^{MC}}{\partial\delta}<0$, indicating that the manufacturer’s carbon reduction rate decreases as the share of green advertising costs increases.

The impact of the manufacturer’s share of green advertising costs $\delta$ on green advertising efforts can be expressed as $\frac{\partial\gamma^{MC}}{\partial\delta}=\frac{k\left( a-P_{c} \right)z\left( k\left( 4v\left( 1-\delta\right)^{2}-z^{2} \right)-\left( P_{c}+t \right)^{2}v\left( 1-\delta\right)^{2} \right)}{\left( 4kv\left( -1+\delta\right)^{2}-\left( P_{c}+t \right)^{2}v\left( -1+\delta\right)^{2}+kz^{2}\left( -2+3\delta\right) \right)^{2}}$. The sign of $\frac{\partial\gamma^{MC}}{\partial\delta}$ is consistent with the sign of $k\left( 4v\left( 1-\delta\right)^{2}-z^{2} \right)-\left( P_{c}+t \right)^{2}v\left( 1-\delta\right)^{2}$.

The impact of the manufacturer's share of green advertising costs $\delta$ on the manufacturer's profit can be expressed as $\frac{\partial\pi_{m}^{MC}}{\partial\delta}=\frac{k^{2}\left( a-P_{c} \right)^{2}vz^{2}\left( 1-\delta\right)\left( 1-3\delta\right)}{2\left( kz^{2}\left( 2-3\delta\right)-4kv\left( -1+\delta\right)^{2}+\left( P_{c}+t \right)^{2}v\left( -1+\delta\right)^{2} \right)^{2}}$. The sign of $\frac{\partial\pi_{m}^{MC}}{\partial\delta}$ is related to $\left( 1-3\delta\right)$. Thus, it is necessary to determine the sign of $\left( 1-3\delta\right)$. When $\left( 1-3\delta\right)>0$, i.e., $\delta\leq\frac{1}{3}$, then $\frac{\partial\pi_{m}^{MC}}{\partial\delta}>0$, indicating that the manufacturer's profit increases as the share of green advertising costs increases. Conversely, when $\left( 1-3\delta\right)<0$, i.e., $\delta>\frac{1}{3}$, then $\frac{\partial\pi_{m}^{MC}}{\partial\delta}<0$, indicating that the manufacturer's profit decreases as the share of green advertising costs increases.

The impact of the manufacturer's share of green advertising costs $\delta$ on the retailer's profit can be expressed as $\frac{\partial\pi_{r}^{MC}}{\partial\delta}=\frac{k^{2}\left( a-P_{c} \right)^{2}vz^{2}\left( -1+\delta\right)^{2}\left( \left( P_{c}+t \right)^{2}v-2\left( 4k+\left( P_{c}+t \right)^{2} \right)v\delta+3kz^{2}\delta+\left( 8k+\left( P_{c}+t \right)^{2} \right)v\delta^{2} \right)}{2\left( 4kv\left( -1+\delta\right)^{2}-\left( P_{c}+t \right)^{2}v\left( -1+\delta\right)^{2}+kz^{2}\left( -2+3\delta\right) \right)^{3}}$. The sign of $\frac{\partial\pi_{r}^{MC}}{\partial\delta}$ is consistent with the sign of $\left( P_{c}+t \right)^{2}v-2\left( 4k+\left( P_{c}+t \right)^{2} \right)v\delta+3kz^{2}\delta+\left( 8k+\left( P_{c}+t \right)^{2} \right)v\delta^{2}$.

*Proof of Comparative analysis*

The comparison of manufacturer profits under different models is shown as follows. Since $\pi_{m}^{MC}-\pi_{m}^{DC}=\frac{k^{2}\left( a-P_{c} \right)^{2}vz^{2}\delta\left( 1-2\delta\right)}{2N\left( k\left( 4v-2z^{2} \right)-\left( P_{c}+t \right)^{2}v \right)}$, and if $\delta^{m}=\frac{1}{2}$, when $\delta<\delta^{m}$, then $\pi_{m}^{MC}>\pi_{m}^{DC}$; otherwise, $\pi_{m}^{MC}<\pi_{m}^{DC}$. Additionally, $\pi_{m}^{RC}-\pi_{m}^{DC}=\frac{k\left( a-P_{c} \right)^{2}\left( P_{c}+t \right)^{2}v^{2}\theta}{2M\left( k\left( 4v-2z^{2} \right)-\left( P_{c}+t \right)^{2}v \right)}>0$; when $\theta<\theta^{h}$, it follows that $\pi_{m}^{RC}>\pi_{m}^{DC}$. Similarly, $\pi_{m}^{MC}-\pi_{m}^{RC}=\frac{{\left( -1+\delta\right)^{2}(a}^{2}kv-2akP_{c}v+kP_{c}^{2}v)}{2N}-\frac{\left( 1-\theta\right)(kP_{c}^{2}v+a^{2}kv-2akP_{c}v)}{2M}$. If $\frac{a^{2}kv\left( -1+\delta\right)^{2}-2akP_{c}v\left( -1+\delta\right)^{2}+kP_{c}^{2}v\left( -1+\delta\right)^{2}}{8kv\left( -1+\delta\right)^{2}-2\left( P_{c}+t \right)^{2}v\left( -1+\delta\right)^{2}+2kz^{2}\left( -2+3\delta\right)}>\frac{kP_{c}^{2}v\left( 1-\theta\right)+a^{2}kv\left( 1-\theta\right)-2akP_{c}v(1-\theta)}{4k\left( 2v-z^{2} \right)\left( 1-\theta\right)-2\left( P_{c}+t \right)^{2}v}$ then $\theta^{m}=1-\frac{\left( P_{c}+t \right)^{2}v\left( -1+\delta\right)^{2}}{2k\left( 2v-z^{2} \right)\left( -1+\delta\right)^{2}-\left( 4kv-\left( P_{c}+t \right)^{2}v \right)\left( -1+\delta\right)^{2}+kz^{2}\left( 2-3\delta\right)}$. If $\theta<\theta^{m}$, then $\pi_{m}^{MC}>\pi_{m}^{RC}$; otherwise, $\pi_{m}^{MC}<\pi_{m}^{RC}$.

The comparison of retailer profits under different models is presented as follows. Since $\pi_{r}^{MC}-\pi_{r}^{DC}=\frac{1}{2}k^{2}\left( a-P_{c} \right)^{2}v(\frac{-2v+z^{2}}{\left( 2k\left( 2v-z^{2} \right)-\left( P_{c}+t \right)^{2}v \right)^{2}}+\frac{(z^{2}+2v\left( -1+\delta\right))\left( -1+\delta\right)^{3}}{N^{2}})$ depends on the inequality $\frac{-2v+z^{2}}{\left( 2k\left( 2v-z^{2} \right)-\left( P_{c}+t \right)^{2}v \right)^{2}}>\frac{(z^{2}+2v\left( -1+\delta\right))\left( 1-\delta\right)^{3}}{\left( kz^{2}\left( 2-3\delta\right)-4kv\left( -1+\delta\right)^{2}+\left( P_{c}+t \right)^{2}v\left( -1+\delta\right)^{2} \right)^{2}}$, we find that if $\delta<\delta^{r}$, then $\pi_{r}^{MC}>\pi_{r}^{DC}$; otherwise, $\pi_{r}^{MC}<\pi_{r}^{DC}$. Additionally, for $\pi_{r}^{RC}-\pi_{r}^{DC}=\frac{1}{2}k\left( a-P_{c} \right)^{2}v(\frac{k\left( -2v+z^{2} \right)}{\left( \left( P_{c}+t \right)^{2}v+2k\left( -2v+z^{2} \right) \right)^{2}}+\frac{k\left( 2v-z^{2} \right)\left( -1+\theta\right)^{2}-\left( P_{c}+t \right)^{2}v\theta}{M^{2}})$, using the condition $\theta_{1}^{r}=-\frac{\left( P_{c}+t \right)^{2}v(\left( P_{c}+t \right)^{2}v+2k(-2v+z^{2}))}{k\left( 2v-z^{2} \right)\left( -\left( P_{c}+t \right)^{2}v+k\left( 8v-4z^{2} \right) \right)}$, it can be stated that if $\theta<\theta^{h}$ and $\theta<\theta_{1}^{r}$, then $\pi_{r}^{RC}>\pi_{r}^{DC}$; otherwise, $\pi_{r}^{RC}<\pi_{r}^{DC}$. Similarly, the comparison $\pi_{r}^{MC}-\pi_{r}^{RC}=\frac{1}{2}k\left( a-P_{c} \right)^{2}v(\frac{k(z^{2}-2v\left( 1-\delta\right))\left( -1+\delta\right)^{3}}{N^{2}}-\frac{k(2v-z^{2})\left( 1-\theta\right)^{2}-\left( P_{c}+t \right)^{2}v\theta}{M^{2}})$ depends on whether $\frac{k(z^{2}-2v\left( 1-\delta\right))\left( -1+\delta\right)^{3}}{\left( kz^{2}\left( 2-3\delta\right)-4kv\left( -1+\delta\right)^{2}+\left( P_{c}+t \right)^{2}v\left( -1+\delta\right)^{2} \right)^{2}}>\frac{k\left( 2v-z^{2} \right)\left( 1-\theta\right)^{2}-\left( P_{c}+t \right)^{2}v\theta}{\left( \left( P_{c}+t \right)^{2}v+2k\left( 2v-z^{2} \right)\left( -1+\theta\right) \right)^{2}}$. If this condition holds when $\theta<\theta_{2}^{r}$ or $\theta<\theta_{3}^{r}$, then $\pi_{r}^{MC}>\pi_{r}^{RC}$; otherwise, $\pi_{r}^{MC}<\pi_{r}^{RC}$. Here, $N=4kv\left( -1+\delta\right)^{2}-\left( P_{c}+t \right)^{2}v\left( -1+\delta\right)^{2}+kz^{2}\left( -2+3\delta\right)$ and $M=2k\left( 2v-z^{2} \right)\left( 1-\theta\right)-\left( P_{c}+t \right)^{2}v$.

The comparison of total supply chain profits under different models is presented as follows. Since $\pi^{MC}-\pi^{DC}=\frac{\begin{aligned} a^{2}kv\left( -1+\delta\right)^{2}-2akP_{c}v\left( -1+\delta\right)^{2}+P_{c}(kP_{c}v\left( -1+\delta\right)^{2}+ \\ 2E_{g}(4kv\left( -1+\delta\right)^{2}-\left( P_{c}+t \right)^{2}v\left( -1+\delta\right)^{2}+kz^{2}(-2+3\delta))) \end{aligned}}{8kv\left( -1+\delta\right)^{2}-2\left( P_{c}+t \right)^{2}v\left( -1+\delta\right)^{2}+2kz^{2}\left( -2+3\delta\right)}+\frac{k^{2}{(a-P_{c})}^{2}v(z^{2}+2v(-1+\delta)){(-1+\delta)}^{3}}{2{(kz^{2}(2-3\delta)-4kv{(-1+\delta)}^{2}+{(P_{c}+t)}^{2}v{(-1+\delta)}^{2})}^{2}}-(\frac{kv(a-P_{c})^{2}+2P_{c}E_{g}\zeta}{2\zeta}+\frac{k^{2}v(a-P_{c})^{2}(2v-z^{2})}{2\zeta^{2}})$, if $\delta<\delta^{s}$, then $\pi^{MC}>\pi^{DC}$; otherwise, $\pi^{MC}<\pi^{DC}$. Additionally, for $\pi^{RC}-\pi^{DC}=\frac{P_{c}(2E_{g}(2\theta k\left( 2v-z^{2} \right)-\zeta)+kP_{c}v(-1+\theta))+kv(-1+\theta)(a^{2}-2aP_{c})}{2(2\theta k\left( 2v-z^{2} \right)-\zeta)}+\frac{k{(a-P_{c})}^{2}v(k(2v-z^{2}){(-1+\theta)}^{2}-{(P_{c}+t)}^{2}v\theta)}{2{(2\theta k\left( 2v-z^{2} \right)-\zeta)}^{2}}-(\frac{kv(a-P_{c})^{2}+2P_{c}E_{g}\zeta}{2\zeta}+\frac{k^{2}v(a-P_{c})^{2}(2v-z^{2})}{2\zeta^{2}})$, if $\theta<\theta_{1}^{s}$, then $\pi^{RC}>\pi^{DC}$; otherwise, $\pi^{RC}<\pi^{DC}$. Similarly, $\pi^{MC}-\pi^{RC}=\frac{a^{2}kv\left( -1+\delta\right)^{2}-2akP_{c}v\left( -1+\delta\right)^{2}+P_{c}(kP_{c}v\left( -1+\delta\right)^{2}+2E_{g}(4kv\left( -1+\delta\right)^{2}-\left( P_{c}+t \right)^{2}v\left( -1+\delta\right)^{2}+kz^{2}(-2+3\delta)))}{8kv\left( -1+\delta\right)^{2}-2\left( P_{c}+t \right)^{2}v\left( -1+\delta\right)^{2}+2kz^{2}\left( -2+3\delta\right)}+\frac{k^{2}\left( a-P_{c} \right)^{2}v\left( z^{2}+2v\left( -1+\delta\right) \right)\left( -1+\delta\right)^{3}}{2\left( kz^{2}\left( 2-3\delta\right)-4kv\left( -1+\delta\right)^{2}+\left( P_{c}+t \right)^{2}v\left( -1+\delta\right)^{2} \right)^{2}}-(\frac{P_{c}(2E_{g}(2\theta k\left( 2v-z^{2} \right)-\zeta)+kP_{c}v(-1+\theta))+kv(-1+\theta)(a^{2}-2aP_{c})}{2(2\theta k\left( 2v-z^{2} \right)-\zeta)}+\frac{k\left( a-P_{c} \right)^{2}v\left( k\left( 2v-z^{2} \right)\left( -1+\theta\right)^{2}-\left( P_{c}+t \right)^{2}v\theta\right)}{2\left( 2\theta k\left( 2v-z^{2} \right)-\zeta\right)^{2}})$ shows that if $\theta<\theta_{2}^{s}$ or $\theta<\theta_{3}^{s}$, then $\pi^{MC}>\pi^{RC}$; otherwise, $\pi^{MC}<\pi^{RC}$. In these expressions, $\zeta=2k\left( 2v-z^{2} \right)-v\left( t+P_{c} \right)^{2}$. Therefore, $\left( P_{c}+t \right)^{2}v+2k\left( 2v-z^{2} \right)\left( -1+\theta\right)=2\theta k\left( 2v-z^{2} \right)-\zeta$.
