# Supplementary material for: Strategies for carbon reduction and advertising investments in partially centralized supply chains
Source: PLoS One. 2026 Jun 16;21(6):e0351412. doi: 10.1371/journal.pone.0351412 (PMC13271493; doi:10.1371/journal.pone.0351412)
Supplement: S1 File — (ZIP) [file pone.0351412.s002.zip › simulation code/parameters.docx]

| Parameter | Definition description | Parameter values |
| --- | --- | --- |
| $a$ | Potential market demand | 50 |
| $t$ | Consumer low-carbon preference | 45 |
| $z$ | Advertising effectiveness | 70 |
| $k$ | Cost coefficient for emission reduction efforts | 5500 |
| $v$ | Cost coefficient for advertising efforts | 5500 |
| $P_{c}$ | Unit carbon trading price | 15 |
| $E_{g}$ | Initial carbon emission quota | 50 |
