# Supplementary material for: Strategies for carbon reduction and advertising investments in partially centralized supply chains
Source: PLoS One. 2026 Jun 16;21(6):e0351412. doi: 10.1371/journal.pone.0351412 (PMC13271493; doi:10.1371/journal.pone.0351412)
Supplement: Attachment — Submitted filename: Reviewers Comments.docx [file pone.0351412.s003.docx]

**Reviewer Comments for Manuscript PONE-D-25-40047**

**Title:** Strategies for Carbon Reduction and Advertising Investments in Partially Centralized Supply Chains

**General Assessment:**
The manuscript addresses an important and timely topic, exploring the interplay between carbon reduction efforts, green advertising, and cost-sharing contracts in supply chains under cap-and-trade policies. The study is theoretically grounded and provides valuable insights into supply chain coordination mechanisms. However, there are several areas where the manuscript could be improved to enhance clarity, rigor, and practical relevance.

**Major Comments:**

1. **Clarity of Model Assumptions and Justifications:**
   - The assumptions underlying the demand function (e.g., linearity in price, carbon reduction rate, and advertising efforts) should be more thoroughly justified. Are there empirical studies or real-world data supporting these assumptions? If not, the authors should discuss the limitations of these simplifications.
   - The cost functions for carbon reduction $\left( \frac{ke^{2}}{2} \right)$ and green advertising ($\frac{v\gamma^{2}}{2}$) are standard in the literature but may not capture real-world complexities. The authors should justify these choices and discuss potential alternatives (e.g., non-quadratic costs).
2. **Empirical Validation:**
   - While the numerical simulations are useful, the manuscript would benefit from empirical validation or case studies to demonstrate the practical applicability of the findings. For instance, could the authors provide examples of firms or industries where the proposed models align with observed behavior?
3. **Policy and Managerial Implications:**
   - The discussion on policy implications is somewhat limited. How might governments design cap-and-trade policies to incentivize the optimal cost-sharing ratios identified in the study?
   - The managerial insights could be expanded to provide clearer guidance for supply chain managers. For example, what specific steps should managers take to determine the most suitable cost-sharing model for their context?
4. **Comparative Analysis of Models:**
   - The comparison between the RC, MC, and DC models is a strength of the study, but the thresholds (e.g., $\theta^{h}, \delta^{m}$) are not intuitively explained. The authors should provide more intuitive interpretations of these thresholds and their practical significance.
5. **Dynamic Considerations:**
   - The study assumes static conditions, but carbon trading prices and consumer preferences are dynamic in reality. How might the findings change if dynamic or stochastic elements were incorporated? This could be a valuable direction for future research.

**Minor Comments:**

1. **Writing and Organization:**
   - The manuscript is well-structured but could benefit from tighter editing for clarity. Some sentences are overly complex (e.g., in the abstract and introduction).
   - The figures (e.g., Figures 3–10) are informative but could be improved with clearer labels.
2. **Literature Review:**
   - The literature review is comprehensive but could better highlight the novel contributions of this study relative to prior work. For example, how does this study advance beyond the findings of [3], [28], or other cited papers?
3. **Parameter Sensitivity:**
   - The sensitivity of results to parameter choices (e.g.,$k, v, P_{c}$) should be discussed further. Are the findings robust to variations in these parameters, or do they hold only under specific conditions?

**Recommendation:**

The manuscript makes a valuable contribution to the literature on green supply chain management and cost-sharing contracts. However, the revisions suggested above would significantly strengthen its theoretical rigor, practical relevance, and clarity. I recommend **major revisions** before acceptance.

**Additional Suggestions:**

- Include a table summarizing the key findings and managerial insights.
- Consider adding a section on limitations and future research directions.
- Ensure all mathematical derivations are clearly presented in the appendices.

Overall, this study has the potential to be a strong addition to the field, and I look forward to seeing the revised version.
