# Supplementary material for: Strategies for carbon reduction and advertising investments in partially centralized supply chains
Source: PLoS One. 2026 Jun 16;21(6):e0351412. doi: 10.1371/journal.pone.0351412 (PMC13271493; doi:10.1371/journal.pone.0351412)
Supplement: Attachment — Submitted filename: 2025-11-26 Response to reviewers.docx [file pone.0351412.s004.docx]

**Strategies for carbon reduction and advertising investments in partially centralized supply chains**

Dear Editors and Reviewers,

On behalf of my co-authors, I would like to express our gratitude for giving us the opportunity to revise our manuscript (Manuscript ID: PONE-D-25-40047). We would like to thank the editors and reviewers for your meticulous evaluation and valuable feedback on our work. Your constructive comments have played a crucial role in improving the quality of our manuscript. We appreciate your contributions to our research.

We carefully reviewed the reviewers’ comments and made further revisions to the manuscript, ensuring that it meets the journal’s requirements while simplifying it as much as possible. The revised sections have been highlighted in blue. We believe that these changes have effectively addressed all the questions and concerns raised by the reviewers.

Following the editorial recommendation to disregard Reviewer #1’s report, no changes have been made in response to those specific comments. We have focused our revisions on the feedback from Reviewers #2 and #3. Detailed responses can be found on page 2 (for Reviewer #2) and pages 2-5 (for Reviewer #3) of this document.

If any further adjustments or modifications are required, please do not hesitate to contact us. We will do our utmost to meet your requirements.

Once again, we would like to thank you and the reviewers for your hard work and support. We look forward to hearing from you soon.

Yours sincerely,

Corresponding Author

**To Reviewer #2**

| 1 | Comment  1 | Thanks to the respected authors for trying to fill the research gap in the study. There is a concern about the presentation of the article, which has lowered its quality, it is better to try to rewrite the article in accordance with the standards. |
| --- | --- | --- |
|  | Response  1 | Thank you for your constructive suggestion. We sincerely appreciate the reviewer’s recognition of the research value and the recommendation to improve the presentation quality of the manuscript.  In response, we have carefully revised the entire paper to enhance clarity, coherence, and adherence to academic writing standards, as well as to align with the journal’s Guide for Authors. Specifically, we have refined the language throughout, reorganized sections for better logical flow, and standardized the presentation of equations, tables, and figures in accordance with the journal’s guidelines. We believe these revisions have significantly improved the overall readability and quality of the manuscript. |

**To Reviewer #3**

| 1 | Comment  1 | The mathematical verification in this manuscript is incomplete. The Hessian matrix analysis provided in the Appendix, Proof of Corollary 3, only verifies second-order conditions for the manufacturer's profit optimisation but omits the retailer's second-order conditions in both RC and MC scenarios. This incomplete verification raises questions about whether the presented solutions truly represent optimal decisions for both parties in the supply chain. |
| --- | --- | --- |
|  | Response  1 | Thank you for your valuable comment. In response, we have carefully revised the mathematical verification to ensure completeness and rigor.  Specifically, we have added the retailer’s second-order conditions under both the RC and MC scenarios, which were previously omitted. Furthermore, to enhance the robustness of the analysis, we have incorporated the Hessian matrix verification for both the manufacturer and the retailer under the SC and DC scenarios.  These additions ensure that the optimal solutions presented in the manuscript satisfy the second-order sufficient conditions for both parties in all relevant settings.  The detailed derivations and proofs are now provided in the revised Appendix (Proof of Lemma 1–4), as shown on pages 31–35 of the manuscript. |
| 2 | Comment  2 | The model adopts standard assumptions of linear demand and quadratic cost functions in Section 3.2 without justification. While these are common in the game theory literature, the authors should at least briefly explain why these functional forms are appropriate for their context or acknowledge them as limitations. The analysis is also limited to a single manufacturer-retailer dyad without discussion of how competition might affect results. |
|  | Response  2 | Thank you for your insightful comment. We have revised Section 3.2 to clarify the rationale for adopting linear demand and quadratic cost functions. We also acknowledge that the study focuses on a single manufacturer–retailer structure, and this limitation has been noted in the “Research Limitations and Future Directions” section of the conclusion.  The adoption of linear demand and quadratic cost functions, which are widely used in the supply chain and game theory literature, as these functional forms allow for analytical tractability while capturing the essential economic characteristics of market demand and production costs. Specifically, the linear demand function reflects the well-established negative relationship between price and market demand, whereas the quadratic cost function appropriately captures the increasing marginal costs associated with emission reduction and promotional efforts. These assumptions help simplify the model, enabling a clearer exploration of strategic interactions and coordination mechanisms between the manufacturer and retailer, which is the primary focus of this study. We believe that these assumptions do not undermine the robustness of our main conclusions but instead facilitate a clearer theoretical exposition.  Regarding the limitation of focusing solely on a single manufacturer-retailer dyad, we acknowledge that the model does not account for competition among multiple manufacturers or retailers. This focus on a dyadic relationship allows for a more in-depth analysis of the key issues involved, but future research may expand the model to include scenarios with multiple manufacturers, multiple retailers, or competitive dynamics, which would offer additional insights.  The updated content can be found on pages 8-9, 28 of the manuscript. |
| 3 | Comment  3 | The finding that high carbon trading prices combined with high reduction cost coefficients may decrease emission reduction efforts (shown in Figure 3) is counterintuitive and important for policy. While the authors provide some economic reasoning about manufacturers shifting strategies, this paradoxical result would benefit from a clearer presentation of the parameter ranges where it occurs and a brief discussion of policy implications. |
|  | Response  3 | Thank you for this insightful comment. In response, we have revised Figure 3 to clarify the parameter ranges in which this counterintuitive result occurs and have added a discussion of the related policy implications in the conclusion section.  Specifically, the updated Figure 3 now highlights the regions where excessively high carbon prices, combined with high emission reduction cost coefficients, lead to a decline in manufacturers’ reduction efforts. This happens because the marginal cost of emission reduction rises sharply, outweighing the potential benefits from carbon trading. To address this counterintuitive finding, we have added a policy discussion emphasizes that overly aggressive carbon pricing may backfire by discouraging firms’ reduction initiatives. Therefore, policymakers should set carbon prices at a moderate and balanced level that aligns environmental objectives with firms’ economic incentives.  These revisions are reflected on pages 18-19 of the manuscript. |
| 4 | Comment  4 | The comparative analysis in Propositions 1-3 provides useful theoretical insights but could benefit from brief discussion of what drives these threshold conditions. For instance, what market characteristics favor the RC model over the MC model? |
|  | Response  4 | Thank you for your comment. In response, we have added a brief discussion to explain the underlying market factors that determine which model RC or MC is more favorable.  Specifically, the manufacturer’s profit primarily depends on the emission reduction cost coefficient and the carbon price. When the manufacturer’s carbon reduction cost is high or the carbon price is moderate, the RC model performs better, as cost sharing by the retailer effectively alleviates the manufacturer’s burden and enhances its incentive to invest in emission reduction. In contrast, under the MC model, the manufacturer bears additional advertising expenses, which weakens its emission reduction motivation. The retailer’s profit is mainly affected by the advertising cost coefficient and consumers’ sensitivity to advertising. When advertising costs are high or consumers respond strongly to green advertising, the MC model is more advantageous because the manufacturer’s cost sharing encourages the retailer to intensify promotional efforts and expand demand. However, when consumers are more concerned about low-carbon attributes, the RC model becomes more favorable as the manufacturer’s stronger carbon reduction investment indirectly enhances product attractiveness.  From a supply chain perspective, the total profit reflects a trade-off between emission reduction efficiency and market expansion. When consumers have strong low-carbon preferences and the carbon price is high, the RC model maximizes supply chain profit; conversely, when consumers are more sensitive to advertising than to emission reduction, the MC model performs better. Overall, the relative superiority of each model depends on the joint influence of market characteristics and policy factors such as the carbon price.  These revisions are reflected on pages 25-28 of the manuscript. |
| 5 | Comment  5 | Regarding data availability, the statement "Data will be made available on request" does not meet PLOS ONE policy, which requires that all data necessary to replicate the study be publicly available at publication. Please deposit the simulation code and parameters in a public repository or include them as Supporting Information, and update the Data Availability Statement accordingly. While not required, providing full simulation scripts would substantially strengthen reproducibility. Additionally, Table 1 is incomplete, missing definitions for profit notation (πm, πr, π) and threshold parameters (δm, δr, θh, θm, θr¹, θr³) that appear in later propositions. Please complete these definitions. Also, the terminology "grandfather law" should be corrected to "grandfathering provisions" throughout the manuscript. |
|  | Response  5 | Thank you for your insightful comment. We have made the corresponding revisions in the paper.  Following the suggestion, we have revised the Data Availability Statement to ensure full compliance with the PLOS ONE policy. Specifically, we have attached all simulation codes, parameters, and related data as supporting information and have updated the data availability statement accordingly.  We have also updated Table 1 to include the missing definitions for the profit notations ($\pi_{m}$, $\pi_{r}$, $\pi$) and threshold parameters ($\delta_{m}$, $\delta_{r}$, $\theta_{h}$, $\theta_{m}$, $\theta_{r}^{1}$, $\theta_{r}^{3}$), ensuring consistency with the propositions in subsequent sections. Furthermore, the terminology “grandfather law” has been corrected to “grandfathering provisions” throughout the manuscript as suggested.  These revisions are reflected on pages 7, 10, 30 of the manuscript. |
