# Supplementary material for: Strategies for carbon reduction and advertising investments in partially centralized supply chains
Source: PLoS One. 2026 Jun 16;21(6):e0351412. doi: 10.1371/journal.pone.0351412 (PMC13271493; doi:10.1371/journal.pone.0351412)
Supplement: Attachment — Submitted filename: 2026-05-02 Response to Comments R2.docx [file pone.0351412.s005.docx]

**Strategies for carbon reduction and advertising investments in partially centralized supply chains**

Dear Editors and Reviewers,

On behalf of my co-authors, I would like to express our gratitude for giving us the opportunity to revise our manuscript (Manuscript ID: PONE-D-25-40047). We would like to thank the editors and reviewers for your meticulous evaluation and valuable feedback on our work. Your constructive comments have played a crucial role in improving the quality of our manuscript. We appreciate your contributions to our research.

We carefully reviewed the reviewers’ comments and made further revisions to the manuscript, ensuring that it meets the journal’s requirements while simplifying it as much as possible. The revised sections have been highlighted in blue. We believe that these changes have effectively addressed all the questions and concerns raised by the reviewers.

Following the editorial recommendation to disregard Reviewer #1’s report, no changes have been made in response to those specific comments. We have focused our revisions on the feedback from Reviewers #4 and #5. Detailed responses can be found on page 2-4 (for Reviewer #4) and pages 5-8 (for Reviewer #5) of this document.

If any further adjustments or modifications are required, please do not hesitate to contact us. We will do our utmost to meet your requirements.

Once again, we would like to thank you and the reviewers for your hard work and support. We look forward to hearing from you soon.

Yours sincerely,

Corresponding Author

**Reviewer #4**: In this paper, the authors introduce four models that analyze different supply chain models with real life examples which is praiseworthy. This approach is distinct from other papers on cost-sharing mechanisms, as it explores a partially centralized model. Furthermore, applicable conditions for the models are discussed that provides a clear understanding of practical application. Following feedbacks may be helpful to improve the manuscript:

1. On page 33, regarding the Hessian matrix of the manufacturer's profit, could the author clarify how they conclude on the non-negativity of the numerator?

Response: Thank you for your careful reading and helpful comment. We acknowledge that, in the original manuscript, the justification for the non-negativity of the numerator in the Hessian matrix of the manufacturer’s profit function was presented in a highly condensed manner, and the intermediate explanation was omitted for brevity.

In the revised manuscript, we have strengthened this part by explicitly adding the missing explanation to clarify the underlying reasoning. Specifically, we now provide a brief statement to indicate why the numerator is guaranteed to be non-negative under the maintained parameter assumptions, thereby improving the transparency and completeness of the derivation.

Changes made in manuscript: We have revised the corresponding part on Page 33 by inserting a clarifying sentence (highlighted in blue) to explicitly explain the non-negativity of the numerator in the Hessian matrix.

2. The author focuses on two types of cost-sharing mechanism (i.e., RC and MC). It would be appreciated if the author could mention some of the challenges of these mechanisms.

Response: Thank you for this insightful suggestion. We agree that discussing the potential challenges associated with the RC and MC cost-sharing mechanisms can further enhance the practical relevance and completeness of the study.

In the revised manuscript, we have incorporated a more detailed discussion of the limitations of these mechanisms in the “Limitations and Future Research” section. Specifically, we now highlight several implementation challenges, including information asymmetry in effort levels that may induce free-riding behavior, potential incentive misalignment between cost-sharing and decision control, and the increased complexity of contract design and coordination. By situating this discussion within the limitations section, we also connect these challenges to potential future research directions, such as exploring alternative or hybrid coordination mechanisms.

Changes made in manuscript: We have revised the “Conclusion” section by adding a new discussion on the practical challenges of RC and MC cost-sharing mechanisms and their implications for future research.

3. This study conducted numerical simulations for four models (e.g., centralized, decentralized, and two partially centralized models) based on assumed parametric values. How do these parametric values change or react to different market conditions?

Response: Thank you for this insightful comment. We agree that examining how parameter values respond to different market conditions is important for assessing the robustness of the model results.

In the revised manuscript, we have extended the numerical analysis by incorporating an additional scenario with a higher carbon trading price. This allows us to investigate how changes in key parameters, particularly carbon pricing, affect supply chain decisions and coordination strategies.

The results show that while variations in carbon price influence the magnitude of equilibrium outcomes, the overall pattern of model selection remains largely unchanged. This indicates that the main qualitative insights of the study are robust across different market conditions.

Changes made in manuscript: We have revised the analysis related to Proposition 3 and Fig. 10 (highlighted in blue) by adding a higher carbon price scenario and discussing the robustness of the results under different parameter settings.

4. There are several repeating statements in the abstract, introduction, and conclusion. Revise or rephrase these sections to improve the readability of the manuscript and maintain a clear and focused narrative.

Response: Thank you for this helpful comment. We agree that some statements were repetitive across the abstract, introduction, and conclusion, which may affect the readability and overall coherence of the manuscript.

In the revised version, we have carefully streamlined these sections to eliminate redundant expressions and improve the clarity of the narrative. Specifically, we have reduced repetitive statements of key findings, refined the structure of the abstract to present more concise insights, and reorganized the conclusion to avoid duplication while enhancing logical flow. We also ensured that each section emphasizes different aspects of the study (i.e., motivation in the introduction, key results in the abstract, and broader implications in the conclusion).

Changes made in manuscript: We have revised the Abstract and Conclusion by removing repetitive statements, rephrasing overlapping content, and improving the overall readability and coherence of the manuscript.

5. In the bibliography, it is evident that no recent (since 2024 to date) paper has been cited. I recommend to cite recent relevant articles, particularly from 2024 to 2026.

Response: Thank you for this valuable suggestion. We agree that incorporating more recent studies can further strengthen the relevance and timeliness of the manuscript.

In the revised manuscript, we have updated the literature review by incorporating recent publications across all major research streams considered in this study. Specifically, we have added new references related to consumer green preferences, green supply chain decisions, and cost-sharing contracts. We have also included recent studies in the assumptions section to further support the modeling framework.

These additions ensure that the manuscript is better aligned with the latest developments in the field and provide a more up-to-date foundation for our analysis.

Changes made in manuscript: We have revised the literature review and Section 3.2 (Assumptions) by adding recent references, which are highlighted in blue throughout the manuscript.

*References added:*

Schulze M, Jürkenbeck K. The Symbiotic Production of Food and Green Electricity: Consumer Preferences for Food Produced in Agrivoltaic Systems. Bus Strategy Environ. 2025;34: 2088–2102. doi:10.1002/bse.4080.

Hsieh C-C, Wu C-Y, Lathifah A. Cross-channel influence of blockchain technology on green supply chains under asymmetric retail platform competition. Int J Prod Econ. 2025;283: 109584. doi:10.1016/j.ijpe.2025.109584.

Cao C, He Y, Liu Y, Huang H, Zhang F. Blockchain technology adoption mechanism for semiconductor chip supply chains considering information disclosure under cost-sharing contract. Int J Prod Econ. 2025;282: 109496. doi:10.1016/j.ijpe.2024.109496.

Zhang W, Mao Q, Pan X, Luo R, Liang L. Two-stage hybrid electricity market trading strategy optimization with renewable electricity under cap-and-trade policy. Energy Econ. 2025;150: 108764. doi:10.1016/j.eneco.2025.108764.

Zhang C, Fan Y, Fang C. Interplay of carbon pricing and emission policies: A game-theoretic study of China’s electricity market. Energy. 2026;345: 140103. doi:10.1016/j.energy.2026.140103.

**Reviewer #5**: This manuscript studies carbon reduction and green advertising decisions in a supply chain under cap-and-trade regulation, considering different cost-sharing mechanisms. The topic is relevant, and the paper provides a structured analytical model with some potentially useful managerial insights. The framework is generally clear, and the combination of carbon reduction and advertising decisions is meaningful. However, there are several issues that should be addressed before the paper can be considered for publication.

Overall Recommendation: The paper addresses a relevant topic and presents a coherent analytical model. With revisions to improve clarity, positioning, and interpretation, it could be suitable for publication.

Recommendation: Minor Revision

1. The paper states that existing studies have not systematically explored the interaction between different supply chain structures and emission reduction strategies. However, there is already a substantial literature on cost-sharing contracts, emission reduction, and supply chain coordination. The current wording somewhat overstates the gap. It would be better to moderate the claim and clarify that prior work has partially addressed these issues, but not in the specific setting considered here (e.g., combining cap-and-trade, green advertising, and partial centralization). A more careful positioning would improve the credibility of the contribution.

Response: Thank you for this insightful comment. We agree that the original wording may overstate the research gap and could be refined to better reflect the existing literature.

In the revised manuscript, we have moderated the relevant statements in the research gap section by acknowledging that prior studies have already provided valuable insights into cost-sharing contracts, emission reduction, and supply chain coordination. We now clarify that our contribution lies not in addressing these issues in isolation, but in integrating them within a unified framework that combines cap-and-trade regulation, green advertising, and partially centralized supply chain structures.

In addition, we have revised the literature review summary to better position our work in relation to existing studies. Specifically, we emphasize that prior research has partially explored these dimensions, while our study extends the literature by jointly examining carbon reduction and green advertising decisions under different coordination structures.

Changes made in manuscript: We have revised the research gap section and the literature review summary (highlighted in blue) to moderate the claims regarding the research gap and to more clearly position our contribution relative to the existing literature.

2. Some assumptions are standard but should still be better explained, especially for a broad audience such as PLOS ONE, These assumptions are reasonable, but brief economic intuition or references should be added to improve clarity.

Response: Thank you for this helpful comment. We agree that, although the assumptions adopted in this study are standard in the literature, providing additional explanation and supporting references can improve clarity, especially for a broad audience such as PLOS ONE.

In the revised manuscript, we have strengthened Section 3.2 (Assumptions) by adding brief explanations of the key variables and model components. Specifically, we now clarify the economic meanings of parameters in the demand function (e.g., market demand, price, carbon reduction rate, and green advertising effort) and explain the formulation of carbon trading under the cap-and-trade mechanism. We have also incorporated relevant references to support these assumptions. These revisions improve the transparency and interpretability of the modeling framework.

Changes made in manuscript: We have revised Section 3.2 (Pages 8–9) by adding explanatory sentences and relevant references to justify the key assumptions and improve clarity.

*References added:*

Du S, Hu L, Wang L. Low-carbon supply policies and supply chain performance with carbon concerned demand. Ann Oper Res. 2017;255: 569–590. doi:10.1007/s10479-015-1988-0.

Du S, Hu L, Song M. Production optimization considering environmental performance and preference in the cap-and-trade system. J Clean Prod. 2016;112: 1600–1607. doi:10.1016/j.jclepro.2014.08.086.

Zhang W, Mao Q, Pan X, Luo R, Liang L. Two-stage hybrid electricity market trading strategy optimization with renewable electricity under cap-and-trade policy. Energy Econ. 2025;150: 108764. doi:10.1016/j.eneco.2025.108764.

Zhang C, Fan Y, Fang C. Interplay of carbon pricing and emission policies: A game-theoretic study of China’s electricity market. Energy. 2026;345: 140103. doi:10.1016/j.energy.2026.140103.

3. While the analytical derivations are detailed, the explanation of results is sometimes too long and repetitive. In particular: the economic intuition behind key findings (e.g., non-monotonic effect of carbon price) could be explained more directly. It would help to summarize the main mechanisms more clearly instead of repeating mathematical interpretations.

Response: Thank you for this insightful comment. We agree that some parts of the original manuscript contained overly detailed explanations and repetitive descriptions of intermediate reasoning, which may obscure the underlying economic intuition.

In the revised manuscript, we have systematically refined the discussion of the numerical results (Figs. 3–7) by streamlining the explanations and focusing more directly on the key economic mechanisms. Specifically, we have reduced step-by-step behavioral descriptions and instead summarized the main insights in terms of concise relationships, such as non-monotonic effects, incentive trade-offs, and the interaction between cost-sharing structures and market responsiveness.

For example, we now highlight the trade-off between emission reduction incentives and cost burdens in explaining the non-monotonic impact of carbon prices (Fig. 3), the role of financial constraints in shaping cost-sharing outcomes (Fig. 4), and the joint effects of consumer sensitivity and coordination mechanisms on profits and advertising decisions (Figs. 5–7). These revisions improve clarity and make the economic intuition more transparent and accessible.

Changes made in manuscript: We have revised the discussions corresponding to Figs. 3–7 by removing redundant descriptions, simplifying the exposition, and clearly summarizing the key economic mechanisms underlying the results.

4. The conclusions provide reasonable insights, but they remain relatively broad. The paper could be improved by: Linking specific findings to concrete managerial decisions and explaining under what conditions each cost-sharing model should be used in practice.

Response: Thank you for this valuable suggestion. We agree that the managerial implications in the original manuscript were relatively broad and could be further strengthened by linking specific findings to concrete decision-making guidelines.

In the revised manuscript, we have refined the managerial insights by moving beyond a direct restatement of model results and instead translating them into higher-level decision guidelines. Specifically, rather than repeating the conditions under which each cost-sharing model is optimal, we now provide a decision-oriented framework that helps managers evaluate carbon price levels, cost structures, and market characteristics when selecting coordination strategies. This revision enhances both the practical relevance and the readability of the manuscript.

Changes made in manuscript: We have revised the managerial implications section in the Conclusion (highlighted in blue) by adding more condition-based and actionable recommendations, and by explicitly linking model results to practical coordination decisions.

5. Some related paper are listed for your consideration:

A. ow-carbon supply policies and supply chain performance with carbon concerned demand

B. roduction optimization considering environmental performance and preference in the cap-and-trade system

Response: Thank you for your helpful suggestion. We appreciate the reviewer for recommending these relevant studies.

In the revised manuscript, we have incorporated the suggested references into Section 3.2 (Assumptions). Specifically, these references are used to support the modeling assumptions and provide additional theoretical grounding for the parameter settings and functional forms adopted in this study.

Changes made in manuscript: We have added the recommended references in Section 3.2 (Pages 8), where they are cited to support the assumptions and improve the overall rigor of the model (highlighted in blue).

6. Language is generally understandable but would benefit from light editing for clarity and conciseness.

Response: Thank you for your helpful comment. We agree that the clarity and conciseness of the manuscript can be further improved. In the revised version, we have carefully reviewed the entire manuscript and made minor language edits to improve readability, clarity, and overall flow, while preserving the original meaning.

Changes made in manuscript: We have performed a thorough language revision throughout the manuscript to enhance clarity and conciseness.
