# Supplementary material for: Strategies for carbon reduction and advertising investments in partially centralized supply chains
Source: PLoS One. 2026 Jun 16;21(6):e0351412. doi: 10.1371/journal.pone.0351412 (PMC13271493; doi:10.1371/journal.pone.0351412)
Supplement: Peer review history

# Peer review: Decision Letter 0

**DOI:** 10.1371/journal.pone.0351412.r001

**Authors:** Biswajit Sarkar

28 Oct 2025

PONE-D-25-40047

Strategies for carbon reduction and advertising investments in partially centralized supply chains

PLOS ONE

Dear Dr. Kong,

Thank you for submitting your manuscript to PLOS ONE. After careful consideration, we feel that it has merit but does not fully meet PLOS ONE’s publication criteria as it currently stands. Therefore, we invite you to submit a revised version of the manuscript that addresses the points raised during the review process.

**ACADEMIC EDITOR:**  The rejection point is significant by a reviewer. However, I would like to give the authors the opportunity to revise the paper for publication. Thus, the authors must revise properly based on the reviewer's comments.

**Comments from the PLOS Editorial Office** : Upon internal evaluation of the reviews provided, we kindly request you to disregard the reviewer report provided by Reviewer 1. No amendments are required in response to Reviewer 1’s comments. We thank you for your attention to this request.

Please submit your revised manuscript by Dec 11 2025 11:59PM. If you will need more time than this to complete your revisions, please reply to this message or contact the journal office at plosone@plos.org. When you're ready to submit your revision, log on to https://www.editorialmanager.com/pone/ and select the 'Submissions Needing Revision' folder to locate your manuscript file.

Please include the following items when submitting your revised manuscript:

- A rebuttal letter that responds to each point raised by the academic editor and reviewer(s). You should upload this letter as a separate file labeled 'Response to Reviewers'.
- A marked-up copy of your manuscript that highlights changes made to the original version. You should upload this as a separate file labeled 'Revised Manuscript with Track Changes'.
- An unmarked version of your revised paper without tracked changes. You should upload this as a separate file labeled 'Manuscript'.

If you would like to make changes to your financial disclosure, please include your updated statement in your cover letter. Guidelines for resubmitting your figure files are available below the reviewer comments at the end of this letter.

If applicable, we recommend that you deposit your laboratory protocols in protocols.io to enhance the reproducibility of your results. Protocols.io assigns your protocol its own identifier (DOI) so that it can be cited independently in the future. For instructions see: https://journals.plos.org/plosone/s/submission-guidelines#loc-laboratory-protocols. Additionally, PLOS ONE offers an option for publishing peer-reviewed Lab Protocol articles, which describe protocols hosted on protocols.io. Read more information on sharing protocols at https://plos.org/protocols?utm_medium=editorial-email&utm_source=authorletters&utm_campaign=protocols.

We look forward to receiving your revised manuscript.

Kind regards,

Prof. Biswajit Sarkar, Ph.D., Post-doc

Academic Editor

PLOS ONE

Journal Requirements:

When submitting your revision, we need you to address these additional requirements.

1.Please ensure that your manuscript meets PLOS ONE's style requirements, including those for file naming. The PLOS ONE style templates can be found at

https://journals.plos.org/plosone/s/file?id=wjVg/PLOSOne_formatting_sample_main_body.pdf and

https://journals.plos.org/plosone/s/file?id=ba62/PLOSOne_formatting_sample_title_authors_affiliations.pdf

2. PLOS requires an ORCID iD for the corresponding author in Editorial Manager on papers submitted after December 6th, 2016. Please ensure that you have an ORCID iD and that it is validated in Editorial Manager. To do this, go to ‘Update my Information’ (in the upper left-hand corner of the main menu), and click on the Fetch/Validate link next to the ORCID field. This will take you to the ORCID site and allow you to create a new iD or authenticate a pre-existing iD in Editorial Manager.

3. Please note that PLOS One has specific guidelines on code sharing for submissions in which author-generated code underpins the findings in the manuscript. In these cases, we expect all author-generated code to be made available without restrictions upon publication of the work. Please review our guidelines at https://journals.plos.org/plosone/s/materials-and-software-sharing#loc-sharing-code and ensure that your code is shared in a way that follows best practice and facilitates reproducibility and reuse.

4. We note that the grant information you provided in the ‘Funding Information’ and ‘Financial Disclosure’ sections do not match.

When you resubmit, please ensure that you provide the correct grant numbers for the awards you received for your study in the ‘Funding Information’ section.

5. We note that your Data Availability Statement is currently as follows

“All relevant data are within the manuscript and its Supporting Information files.”

Please confirm at this time whether or not your submission contains all raw data required to replicate the results of your study. Authors must share the “minimal data set” for their submission. PLOS defines the minimal data set to consist of the data required to replicate all study findings reported in the article, as well as related metadata and methods (https://journals.plos.org/plosone/s/data-availability#loc-minimal-data-set-definition).

For example, authors should submit the following data:

- The values behind the means, standard deviations and other measures reported;

- The values used to build graphs;

- The points extracted from images for analysis.

Authors do not need to submit their entire data set if only a portion of the data was used in the reported study.

If your submission does not contain these data, please either upload them as Supporting Information files or deposit them to a stable, public repository and provide us with the relevant URLs, DOIs, or accession numbers. For a list of recommended repositories, please see https://journals.plos.org/plosone/s/recommended-repositories.

If there are ethical or legal restrictions on sharing a de-identified data set, please explain them in detail (e.g., data contain potentially sensitive information, data are owned by a third-party organization, etc.) and who has imposed them (e.g., an ethics committee). Please also provide contact information for a data access committee, ethics committee, or other institutional body to which data requests may be sent. If data are owned by a third party, please indicate how others may request data access.

6. We note you have included a table to which you do not refer in the text of your manuscript. Please ensure that you refer to Table 1 in your text; if accepted, production will need this reference to link the reader to the Table.

7. If the reviewer comments include a recommendation to cite specific previously published works, please review and evaluate these publications to determine whether they are relevant and should be cited. There is no requirement to cite these works unless the editor has indicated otherwise.

Additional Editor Comments:

The rejection point is significant by a reviewer. However, I would like to give the authors the opportunity to revise the paper for publication. Thus, the authors must revise properly based on the reviewer's comments.

[Note: HTML markup is below. Please do not edit.]

Reviewer's Responses to Questions

**Comments to the Author**

1. Is the manuscript technically sound, and do the data support the conclusions?

The manuscript must describe a technically sound piece of scientific research with data that supports the conclusions. Experiments must have been conducted rigorously, with appropriate controls, replication, and sample sizes. The conclusions must be drawn appropriately based on the data presented.

Reviewer #1: Yes

Reviewer #2: No

Reviewer #3: Partly

**********

2. Has the statistical analysis been performed appropriately and rigorously?

Reviewer #1: Yes

Reviewer #2: No

Reviewer #3: N/A

**********

3. Have the authors made all data underlying the findings in their manuscript fully available?

The PLOS Data policy requires authors to make all data underlying the findings described in their manuscript fully available without restriction, with rare exception (please refer to the Data Availability Statement in the manuscript PDF file). The data should be provided as part of the manuscript or its supporting information, or deposited to a public repository. For example, in addition to summary statistics, the data points behind means, medians and variance measures should be available. If there are restrictions on publicly sharing data—e.g. participant privacy or use of data from a third party—those must be specified.

Reviewer #1: Yes

Reviewer #2: No

Reviewer #3: No

**********

4. Is the manuscript presented in an intelligible fashion and written in standard English?

PLOS ONE does not copyedit accepted manuscripts, so the language in submitted articles must be clear, correct, and unambiguous. Any typographical or grammatical errors should be corrected at revision, so please note any specific errors here.

Reviewer #1: Yes

Reviewer #2: No

Reviewer #3: Yes

**********

5. Review Comments to the Author

Please use the space provided to explain your answers to the questions above. You may also include additional comments for the author, including concerns about dual publication, research ethics, or publication ethics. (Please upload your review as an attachment if it exceeds 20,000 characters)

Reviewer #1: Reviewer Comments for Manuscript PONE-D-25-40047

Title: Strategies for Carbon Reduction and Advertising Investments in Partially Centralized Supply Chains

General Assessment:

The manuscript addresses an important and timely topic, exploring the interplay between carbon reduction efforts, green advertising, and cost-sharing contracts in supply chains under cap-and-trade policies. The study is theoretically grounded and provides valuable insights into supply chain coordination mechanisms. However, there are several areas where the manuscript could be improved to enhance clarity, rigor, and practical relevance.

Major Comments:

Clarity of Model Assumptions and Justifications:

The assumptions underlying the demand function (e.g., linearity in price, carbon reduction rate, and advertising efforts) should be more thoroughly justified. Are there empirical studies or real-world data supporting these assumptions? If not, the authors should discuss the limitations of these simplifications.

The cost functions for carbon reduction ((ke^2)/2) and green advertising ((vγ^2)/2) are standard in the literature but may not capture real-world complexities. The authors should justify these choices and discuss potential alternatives (e.g., non-quadratic costs).

Empirical Validation:

While the numerical simulations are useful, the manuscript would benefit from empirical validation or case studies to demonstrate the practical applicability of the findings. For instance, could the authors provide examples of firms or industries where the proposed models align with observed behavior?

Policy and Managerial Implications:

The discussion on policy implications is somewhat limited. How might governments design cap-and-trade policies to incentivize the optimal cost-sharing ratios identified in the study?

The managerial insights could be expanded to provide clearer guidance for supply chain managers. For example, what specific steps should managers take to determine the most suitable cost-sharing model for their context?

Comparative Analysis of Models:

The comparison between the RC, MC, and DC models is a strength of the study, but the thresholds (e.g., θ^h,δ^m) are not intuitively explained. The authors should provide more intuitive interpretations of these thresholds and their practical significance.

Dynamic Considerations:

The study assumes static conditions, but carbon trading prices and consumer preferences are dynamic in reality. How might the findings change if dynamic or stochastic elements were incorporated? This could be a valuable direction for future research.

Minor Comments:

Writing and Organization:

The manuscript is well-structured but could benefit from tighter editing for clarity. Some sentences are overly complex (e.g., in the abstract and introduction).

The figures (e.g., Figures 3–10) are informative but could be improved with clearer labels.

Literature Review:

The literature review is comprehensive but could better highlight the novel contributions of this study relative to prior work. For example, how does this study advance beyond the findings of [3], [28], or other cited papers?

Parameter Sensitivity:

The sensitivity of results to parameter choices (e.g., k,v,P_c) should be discussed further. Are the findings robust to variations in these parameters, or do they hold only under specific conditions?

Recommendation:

The manuscript makes a valuable contribution to the literature on green supply chain management and cost-sharing contracts. However, the revisions suggested above would significantly strengthen its theoretical rigor, practical relevance, and clarity. I recommend major revisions before acceptance.

Additional Suggestions:

Include a table summarizing the key findings and managerial insights.

Consider adding a section on limitations and future research directions.

Ensure all mathematical derivations are clearly presented in the appendices.

Overall, this study has the potential to be a strong addition to the field, and I look forward to seeing the revised version.

Reviewer #2: Thanks to the respected authors for trying to fill the research gap in the study. There is a concern about the presentation of the article, which has lowered its quality, it is better to try to rewrite the article in accordance with the standards.

Reviewer #3: This manuscript examines carbon reduction and green advertising strategies in supply chains under a cap-and-trade policy through game theory modelling of four coordination scenarios. While the research addresses an important and timely topic with a generally sound mathematical framework, several critical issues require revision before the manuscript meets publication standards.

The mathematical verification in this manuscript is incomplete. The Hessian matrix analysis provided in the Appendix, Proof of Corollary 3, only verifies second-order conditions for the manufacturer's profit optimisation but omits the retailer's second-order conditions in both RC and MC scenarios. This incomplete verification raises questions about whether the presented solutions truly represent optimal decisions for both parties in the supply chain.

The model adopts standard assumptions of linear demand and quadratic cost functions in Section 3.2 without justification. While these are common in the game theory literature, the authors should at least briefly explain why these functional forms are appropriate for their context or acknowledge them as limitations. The analysis is also limited to a single manufacturer-retailer dyad without discussion of how competition might affect results.

The finding that high carbon trading prices combined with high reduction cost coefficients may decrease emission reduction efforts (shown in Figure 3) is counterintuitive and important for policy. While the authors provide some economic reasoning about manufacturers shifting strategies, this paradoxical result would benefit from a clearer presentation of the parameter ranges where it occurs and a brief discussion of policy implications.

The comparative analysis in Propositions 1-3 provides useful theoretical insights but could benefit from brief discussion of what drives these threshold conditions. For instance, what market characteristics favor the RC model over the MC model?

Regarding data availability, the statement "Data will be made available on request" does not meet PLOS ONE policy, which requires that all data necessary to replicate the study be publicly available at publication. Please deposit the simulation code and parameters in a public repository or include them as Supporting Information, and update the Data Availability Statement accordingly. While not required, providing full simulation scripts would substantially strengthen reproducibility. Additionally, Table 1 is incomplete, missing definitions for profit notation (πm, πr, π) and threshold parameters (δm, δr, θh, θm, θr¹, θr³) that appear in later propositions. Please complete these definitions. Also, the terminology "grandfather law" should be corrected to "grandfathering provisions" throughout the manuscript.

Despite these concerns, the manuscript has notable strengths, including its novel partial centralisation framework comparing RC and MC models, comprehensive analysis across four coordination structures, and integration of both carbon reduction and advertising decisions within a unified framework. The core gam theory approach appears well, and the research questions are relevant to current sustainability challenges in supply chain management.

The manuscript requires major revision to address these fundamental issues. Specifically, the authors must provide complete mathematical verification including all second order conditions for both manufacturer and retailer, add brief justification for key assumptions or acknowledge their limitations, provide clearer presentation of the paradoxical carbon price effect with discussion of when it occurs and its policy implications, and ensure proper data availability by providing simulation code and parameters in supplementary materials or a public repository.

**********

6. PLOS authors have the option to publish the peer review history of their article (what does this mean?). If published, this will include your full peer review and any attached files.

If you choose “no”, your identity will remain anonymous but your review may still be made public.

**Do you want your identity to be public for this peer review?**  For information about this choice, including consent withdrawal, please see our Privacy Policy.

Reviewer #1: **Yes:**  Mohamed A.Wahab ALI

Reviewer #2: No

Reviewer #3: No

**********

[NOTE: If reviewer comments were submitted as an attachment file, they will be attached to this email and accessible via the submission site. Please log into your account, locate the manuscript record, and check for the action link "View Attachments". If this link does not appear, there are no attachment files.]

To ensure your figures meet our technical requirements, please review our figure guidelines: https://journals.plos.org/plosone/s/figures

You may also use PLOS’s free figure tool, NAAS, to help you prepare publication quality figures: https://journals.plos.org/plosone/s/figures#loc-tools-for-figure-preparation.

NAAS will assess whether your figures meet our technical requirements by comparing each figure against our figure specifications.

Attachment Submitted filename: Reviewers Comments.docx

# Author comment: Author response to Decision Letter 1

**DOI:** 10.1371/journal.pone.0351412.r002

8 Dec 2025

Dear Editors and Reviewers,

On behalf of my co-authors, I would like to express our gratitude for giving us the opportunity to revise our manuscript (Manuscript ID: PONE-D-25-40047). We would like to thank the editors and reviewers for your meticulous evaluation and valuable feedback on our work. Your constructive comments have played a crucial role in improving the quality of our manuscript. We appreciate your contributions to our research.

We carefully reviewed the reviewers’ comments and made further revisions to the manuscript, ensuring that it meets the journal’s requirements while simplifying it as much as possible. The revised sections have been highlighted in blue. We believe that these changes have effectively addressed all the questions and concerns raised by the reviewers.

For details, please refer to the Response to Reviewers.

Following the editorial recommendation to disregard Reviewer #1’s report, no changes have been made in response to those specific comments. We have focused our revisions on the feedback from Reviewers #2 and #3. Detailed responses can be found on page 2 (for Reviewer #2) and pages 2-5 (for Reviewer #3) of this document.

If any further adjustments or modifications are required, please do not hesitate to contact us. We will do our utmost to meet your requirements.

Once again, we would like to thank you and the reviewers for your hard work and support. We look forward to hearing from you soon.

Yours sincerely,

Corresponding Author

Attachment Submitted filename: 2025-11-26 Response to reviewers.docx

# Peer review: Decision Letter 1

**DOI:** 10.1371/journal.pone.0351412.r003

**Authors:** João Zambujal-Oliveira

21 Apr 2026

PONE-D-25-40047R1

Strategies for carbon reduction and advertising investments in partially centralized supply chains

PLOS One

Dear Dr. Liu,

Thank you for submitting your manuscript to PLOS ONE. After careful consideration, we feel that it has merit but does not fully meet PLOS ONE’s publication criteria as it currently stands. Therefore, we invite you to submit a revised version of the manuscript that addresses the points raised during the review process.

According to the reviewers’ comments, there are several issues that should be addressed prior to any form of acceptance. Although these were classified as minor revisions, I consider them to be major, as they involve adding citations to the reviewers’ own work, which is not permitted under PLOS policies. As a result, such statements have been removed. If, for any reason, any of them remain, they should be disregarded.

Please submit your revised manuscript by Jun 05 2026 11:59PM. If you will need more time than this to complete your revisions, please reply to this message or contact the journal office at plosone@plos.org. When you're ready to submit your revision, log on to https://www.editorialmanager.com/pone/ and select the 'Submissions Needing Revision' folder to locate your manuscript file.

Please include the following items when submitting your revised manuscript:

- A letter that responds to each point raised by the academic editor and reviewer(s). You should upload this letter as a separate file labeled 'Response to Reviewers'.
- A marked-up copy of your manuscript that highlights changes made to the original version. You should upload this as a separate file labeled 'Revised Manuscript with Track Changes'.
- An unmarked version of your revised paper without tracked changes. You should upload this as a separate file labeled 'Manuscript'.

If you would like to make changes to your financial disclosure, please include your updated statement in your cover letter. Guidelines for resubmitting your figure files are available below the reviewer comments at the end of this letter.

If applicable, we recommend that you deposit your laboratory protocols in protocols.io to enhance the reproducibility of your results. Protocols.io assigns your protocol its own identifier (DOI) so that it can be cited independently in the future. For instructions see: https://journals.plos.org/plosone/s/submission-guidelines#loc-laboratory-protocols. Additionally, PLOS ONE offers an option for publishing peer-reviewed Lab Protocol articles, which describe protocols hosted on protocols.io. Read more information on sharing protocols at https://plos.org/protocols?utm_medium=editorial-email&utm_source=authorletters&utm_campaign=protocols.

As the corresponding author, your ORCID iD is verified in the submission system and will appear in the published article. PLOS supports the use of ORCID, and we encourage all coauthors to register for an ORCID iD and use it as well. Please encourage your coauthors to verify their ORCID iD within the submission system before final acceptance, as unverified ORCID iDs will not appear in the published article. *Only*  the individual author can complete the verification step; PLOS staff *cannot*  verify ORCID iDs on behalf of authors.

We look forward to receiving your revised manuscript.

Kind regards,

João Zambujal-Oliveira

Academic Editor

PLOS One

Journal Requirements:

If the reviewer comments include a recommendation to cite specific previously published works, please review and evaluate these publications to determine whether they are relevant and should be cited. There is no requirement to cite these works unless the editor has indicated otherwise.

[Note: HTML markup is below. Please do not edit.]

Reviewers' comments:

Reviewer's Responses to Questions

**Comments to the Author**

1. If the authors have adequately addressed your comments raised in a previous round of review and you feel that this manuscript is now acceptable for publication, you may indicate that here to bypass the “Comments to the Author” section, enter your conflict of interest statement in the “Confidential to Editor” section, and submit your "Accept" recommendation.

Reviewer #4: All comments have been addressed

Reviewer #5: (No Response)

**********

2. Is the manuscript technically sound, and do the data support the conclusions?

The manuscript must describe a technically sound piece of scientific research with data that supports the conclusions. Experiments must have been conducted rigorously, with appropriate controls, replication, and sample sizes. The conclusions must be drawn appropriately based on the data presented.

Reviewer #4: Yes

Reviewer #5: Yes

**********

3. Has the statistical analysis been performed appropriately and rigorously?

Reviewer #4: N/A

Reviewer #5: N/A

**********

4. Have the authors made all data underlying the findings in their manuscript fully available?

The PLOS Data policy requires authors to make all data underlying the findings described in their manuscript fully available without restriction, with rare exception (please refer to the Data Availability Statement in the manuscript PDF file). The data should be provided as part of the manuscript or its supporting information, or deposited to a public repository. For example, in addition to summary statistics, the data points behind means, medians and variance measures should be available. If there are restrictions on publicly sharing data—e.g. participant privacy or use of data from a third party—those must be specified.

Reviewer #4: Yes

Reviewer #5: No

**********

5. Is the manuscript presented in an intelligible fashion and written in standard English?

PLOS ONE does not copyedit accepted manuscripts, so the language in submitted articles must be clear, correct, and unambiguous. Any typographical or grammatical errors should be corrected at revision, so please note any specific errors here.

Reviewer #4: Yes

Reviewer #5: Yes

**********

6. Review Comments to the Author

Please use the space provided to explain your answers to the questions above. You may also include additional comments for the author, including concerns about dual publication, research ethics, or publication ethics. (Please upload your review as an attachment if it exceeds 20,000 characters)

Reviewer #4: In this paper, the authors introduce four models that analyze different supply chain models with real life examples which is praiseworthy. This approach is distinct from other papers on cost-sharing mechanisms, as it explores a partially centralized model. Furthermore, applicable conditions for the models are discussed that provides a clear understanding of practical application.

Following feedbacks may be helpful to improve the manuscript:

1. On page 33, regarding the Hessian matrix of the manufacturer's profit, could the author clarify how they conclude on the non-negativity of the numerator?

2. The author focuses on two types of cost-sharing mechanism (i.e., RC and MC). It would be appreciated if the author could mention some of the challenges of these mechanisms.

3. This study conducted numerical simulations for four models (e.g., centralized, decentralized, and two partially centralized models) based on assumed parametric values. How do these parametric values change or react to different market conditions?

4. There are several repeating statements in the abstract, introduction, and conclusion. Revise or rephrase these sections to improve the readability of the manuscript and maintain a clear and focused narrative.

5. In the bibliography, it is evident that no recent (since 2024 to date) paper has been cited. I recommend to cite recent relevant articles, particularly from 2024 to 2026.

Reviewer #5: This manuscript studies carbon reduction and green advertising decisions in a supply chain under cap-and-trade regulation, considering different cost-sharing mechanisms. The topic is relevant, and the paper provides a structured analytical model with some potentially useful managerial insights. The framework is generally clear, and the combination of carbon reduction and advertising decisions is meaningful. However, there are several issues that should be addressed before the paper can be considered for publication.

1. The paper states that existing studies have not systematically explored the interaction between different supply chain structures and emission reduction strategies. However, there is already a substantial literature on cost-sharing contracts, emission reduction, and supply chain coordination. The current wording somewhat overstates the gap. It would be better to moderate the claim and clarify that prior work has partially addressed these issues, but not in the specific setting considered here (e.g., combining cap-and-trade, green advertising, and partial centralization). A more careful positioning would improve the credibility of the contribution.

2.Some assumptions are standard but should still be better explained, especially for a broad audience such as PLOS ONE, These assumptions are reasonable, but brief economic intuition or references should be added to improve clarity.

3.While the analytical derivations are detailed, the explanation of results is sometimes too long and repetitive. In particular: the economic intuition behind key findings (e.g., non-monotonic effect of carbon price) could be explained more directly. It would help to summarize the main mechanisms more clearly instead of repeating mathematical interpretations.

4.The conclusions provide reasonable insights, but they remain relatively broad. The paper could be improved by: Linking specific findings to concrete managerial decisions and explaining under what conditions each cost-sharing model should be used in practice.

5.Some related paper are listed for your consideration:

A.Low-carbon supply policies and supply chain performance with carbon concerned demand

B.Production optimization considering environmental performance and preference in the cap-and-trade system

6.Language is generally understandable but would benefit from light editing for clarity and conciseness.

Overall Recommendation: The paper addresses a relevant topic and presents a coherent analytical model. With revisions to improve clarity, positioning, and interpretation, it could be suitable for publication.

Recommendation: Minor Revision

**********

7. PLOS authors have the option to publish the peer review history of their article (what does this mean?). If published, this will include your full peer review and any attached files.

If you choose “no”, your identity will remain anonymous but your review may still be made public.

**Do you want your identity to be public for this peer review?**  For information about this choice, including consent withdrawal, please see our Privacy Policy.

Reviewer #4: **Yes:**  ZIAUL HAQ ADNAN

Reviewer #5: No

**********

[NOTE: If reviewer comments were submitted as an attachment file, they will be attached to this email and accessible via the submission site. Please log into your account, locate the manuscript record, and check for the action link "View Attachments". If this link does not appear, there are no attachment files.]

To ensure your figures meet our technical requirements, please review our figure guidelines: https://journals.plos.org/plosone/s/figures

You may also use PLOS’s free figure tool, NAAS, to help you prepare publication quality figures: https://journals.plos.org/plosone/s/figures#loc-tools-for-figure-preparation.

NAAS will assess whether your figures meet our technical requirements by comparing each figure against our figure specifications.

# Author comment: Author response to Decision Letter 2

**DOI:** 10.1371/journal.pone.0351412.r004

6 May 2026

Thank you very much for your consideration of our revised manuscript. We have carefully addressed the specific comments raised by the reviewers and editors. Please see the attached file “Response to Reviewers” for our detailed point-by-point responses.

Attachment Submitted filename: 2026-05-02 Response to Comments R2.docx

# Peer review: Decision Letter 2

**DOI:** 10.1371/journal.pone.0351412.r005

**Authors:** João Zambujal-Oliveira

27 May 2026

Strategies for carbon reduction and advertising investments in partially centralized supply chains

PONE-D-25-40047R2

Dear Dr. Liu,

We’re pleased to inform you that your manuscript has been judged scientifically suitable for publication and will be formally accepted for publication once it meets all outstanding technical requirements.

Within one week, you’ll receive an e-mail detailing the required amendments. When these have been addressed, you’ll receive a formal acceptance letter and your manuscript will be scheduled for publication.

An invoice will be generated when your article is formally accepted. Please note, if your institution has a publishing partnership with PLOS and your article meets the relevant criteria, all or part of your publication costs will be covered. Please make sure your user information is up-to-date by logging into Editorial Manager at Editorial Manager® and clicking the ‘Update My Information' link at the top of the page. For questions related to billing, please contact billing support.

If your institution or institutions have a press office, please notify them about your upcoming paper to help maximize its impact. If they’ll be preparing press materials, please inform our press team as soon as possible -- no later than 48 hours after receiving the formal acceptance. Your manuscript will remain under strict press embargo until 2 pm Eastern Time on the date of publication. For more information, please contact onepress@plos.org.

Kind regards,

João Zambujal-Oliveira

Academic Editor

PLOS One

Additional Editor Comments (optional):

Reviewers' comments:

Reviewer's Responses to Questions

**Comments to the Author**

1. If the authors have adequately addressed your comments raised in a previous round of review and you feel that this manuscript is now acceptable for publication, you may indicate that here to bypass the “Comments to the Author” section, enter your conflict of interest statement in the “Confidential to Editor” section, and submit your "Accept" recommendation.

Reviewer #4: All comments have been addressed

Reviewer #5: All comments have been addressed

**********

2. Is the manuscript technically sound, and do the data support the conclusions?

The manuscript must describe a technically sound piece of scientific research with data that supports the conclusions. Experiments must have been conducted rigorously, with appropriate controls, replication, and sample sizes. The conclusions must be drawn appropriately based on the data presented.

Reviewer #4: Yes

Reviewer #5: Yes

**********

3. Has the statistical analysis been performed appropriately and rigorously?

Reviewer #4: N/A

Reviewer #5: Yes

**********

4. Have the authors made all data underlying the findings in their manuscript fully available?

The PLOS Data policy requires authors to make all data underlying the findings described in their manuscript fully available without restriction, with rare exception (please refer to the Data Availability Statement in the manuscript PDF file). The data should be provided as part of the manuscript or its supporting information, or deposited to a public repository. For example, in addition to summary statistics, the data points behind means, medians and variance measures should be available. If there are restrictions on publicly sharing data—e.g. participant privacy or use of data from a third party—those must be specified.

Reviewer #4: Yes

Reviewer #5: Yes

**********

5. Is the manuscript presented in an intelligible fashion and written in standard English?

PLOS ONE does not copyedit accepted manuscripts, so the language in submitted articles must be clear, correct, and unambiguous. Any typographical or grammatical errors should be corrected at revision, so please note any specific errors here.

Reviewer #4: Yes

Reviewer #5: Yes

**********

6. Review Comments to the Author

Please use the space provided to explain your answers to the questions above. You may also include additional comments for the author, including concerns about dual publication, research ethics, or publication ethics. (Please upload your review as an attachment if it exceeds 20,000 characters)

Reviewer #4: The author has revised the manuscript in accordance with all my previous comments, effectively addressing each point raised. The revisions have improved the clarity and quality of the manuscript. All concerns have been adequately addressed. I find the responses satisfactory, and there are no major unresolved issues. Therefore, I recommend the manuscript for further editorial consideration and possible acceptance.

Reviewer #5: All my questions have been well addressed. It is recommended that the author carefully review the entire article before the journal accept it.

**********

7. PLOS authors have the option to publish the peer review history of their article (what does this mean?). If published, this will include your full peer review and any attached files.

If you choose “no”, your identity will remain anonymous but your review may still be made public.

**Do you want your identity to be public for this peer review?**  For information about this choice, including consent withdrawal, please see our Privacy Policy.

Reviewer #4: No

Reviewer #5: No

**********

# Editor report: Acceptance letter

**DOI:** 10.1371/journal.pone.0351412.r006

**Authors:** João Zambujal-Oliveira

PONE-D-25-40047R2

PLOS One

Dear Dr. Liu,

I'm pleased to inform you that your manuscript has been deemed suitable for publication in PLOS One. Congratulations! Your manuscript is now being handed over to our production team.

At this stage, our production department will prepare your paper for publication. This includes ensuring the following:

* All references, tables, and figures are properly cited

* All relevant supporting information is included in the manuscript submission,

* There are no issues that prevent the paper from being properly typeset

You will receive further instructions from the production team, including instructions on how to review your proof when it is ready. Please keep in mind that we are working through a large volume of accepted articles, so please give us a few days to review your paper and let you know the next and final steps.

Lastly, if your institution or institutions have a press office, please let them know about your upcoming paper now to help maximize its impact. If they'll be preparing press materials, please inform our press team within the next 48 hours. Your manuscript will remain under strict press embargo until 2 pm Eastern Time on the date of publication. For more information, please contact onepress@plos.org.

You will receive an invoice from PLOS for your publication fee after your manuscript has reached the completed accept phase. If you receive an email requesting payment before acceptance or for any other service, this may be a phishing scheme. Learn how to identify phishing emails and protect your accounts at https://explore.plos.org/phishing.

If we can help with anything else, please email us at customercare@plos.org.

Thank you for submitting your work to PLOS ONE and supporting open access.

Kind regards,

PLOS ONE Editorial Office Staff

on behalf of

Prof. João Zambujal-Oliveira

Academic Editor

PLOS One
